# Synthesis and Evaluation of Glucosyl-, Acyl- and Silyl- Resveratrol Derivatives as Retinoprotective Agents: Piceid Octanoate Notably Delays Photoreceptor Degeneration in a Retinitis Pigmentosa Mouse Model

**DOI:** 10.3390/ph17111482

**Published:** 2024-11-05

**Authors:** Lourdes Valdés-Sánchez, Seyed Mohamadmehdi Moshtaghion, Estefanía Caballano-Infantes, Pablo Peñalver, Rosario Rodríguez-Ruiz, José Luis González-Alfonso, Francisco José Plou, Tom Desmet, Juan C. Morales, Francisco J. Díaz-Corrales

**Affiliations:** 1 Department of Integrative Pathophysiology and Therapies, Andalusian Molecular Biology and Regenerative Medicine Centre (CABIMER), Junta de Andalucía, CSIC, Universidad de Sevilla, Universidad Pablo de Olavide, Avda. Américo Vespucio 24, 41092 Seville, Seville, Spain; lourdes.valdes@cabimer.es (L.V.-S.); mehdi.moshtaghion@cabimer.es (S.M.M.); estefania.caballano@cabimer.es (E.C.-I.); 2 Department of Biochemistry and Molecular Pharmacology, Instituto de Parasitología y Biomedicina López Neyra, CSIC, PTS Granada, Avda. del Conocimiento, 17, 18016 Armilla, Granada, Spain; pablo@ipb.csic.es (P.P.); rosariorodriguezruiz@hotmail.com (R.R.-R.); 3 Instituto de Catálisis y Petroleoquímica, CSIC, Marie Curie 2, 28049 Madrid, Madrid, Spain; josel.g@csic.es (J.L.G.-A.) fplou@icp.csic.es (F.J.P.); 4 Centre for Synthetic Biology, Department of Biotechnology, Ghent University, Coupure Links 653, 9000 Ghent, Belgium; tom.desmet@ugent.be

**Keywords:** neurodegeneration, retinal degeneration, retinitis pigmentosa, resveratrol, piceid octanoate, retinoprotection

## Abstract

Background: Retinitis pigmentosa (RP), the leading cause of inherited blindness in adults, is marked by the progressive degeneration of rod photoreceptors in the retina. While gene therapy has shown promise in treating RP in patients with specific mutations, no effective therapies currently exist for the majority of patients with diverse genetic backgrounds. Additionally, no intervention can yet prevent or delay photoreceptor loss across the broader RP patient population. Resveratrol (RES), a naturally occurring polyphenol, has shown cytoprotective effects in various neurodegenerative disease models; however, its therapeutic potential is limited by low bioavailability. Methods: In this study, we synthesized novel RES derivatives and assessed their retinoprotective effects in a murine model of RP (rd10 mice). Results: Among these derivatives, piceid octanoate (PIC-OCT) significantly delayed photoreceptor degeneration in the RP model, demonstrating superior efficacy compared to RES. Conclusions: PIC-OCT shows strong potential as a leading candidate for developing new therapeutic strategies for RP.

## 1. Introduction

**Figure 1:**
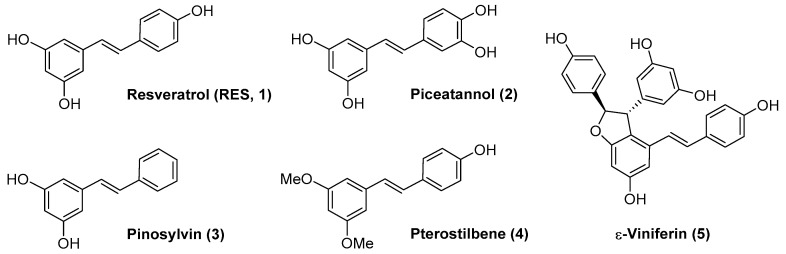
Resveratrol (RES, **1**) and related natural products (compounds **2**–**5**).

Resveratrol (RES) (Figure 1, compound **1**) is a polyphenolic natural product with a stilbene structure. It can be found in several plants such as the Japanese knotweed *Polygonum cuspidatum*, and in food sources such as grapes, nuts, blackberries, peanuts, chocolate, etc. [1]. RES has shown preventive and therapeutic effects on a wide variety of pathologies such as cardiovascular diseases [2,3], cancer [4,5], diabetes [6] or neurodegenerative diseases [7,8,9]. The high number of clinical trials completed and ongoing (over two hundred and over twenty, respectively) reveals the potential of RES as a drug [10].

In the case of ocular diseases, the antioxidant and anti-inflammatory activity of RES has also been reported [11,12,13]. In vitro studies have shown that RES can inhibit hyperglycemia-induced inflammation in retinal pigment epithelium (RPE) cells [14], suppress the expression of vascular endothelial growth factor (VEGF) [15] and protect photoreceptors by blocking caspase- and PARP-dependent cell death pathways [16]. RES as a drug treatment in in vivo models has also been investigated. For example, RES attenuated retinal inflammation and damage on a diabetic retinopathy mice model [17] and ameliorated retinal ganglion cell degeneration [18]. In a neovascular age-related macular degeneration (AMD) mice model, (*Vldlr*^−/−^ mouse), RES inhibited VEGF expression and the angiogenic activation of retinal endothelial cells [19]. Notably, two studies have been accomplished with three patients each, diagnosed with dry AMD or wet AMD, who were treated with daily oral RES as part of a nutritional supplement. In both cases, the authors found the restoration of structure and visual function, together with no side effects after multi-year administration (100 mg/day) [20,21,22].

The results in humans are promising but there are no data yet on a larger study that could reinforce them. Moreover, there is great controversy about the bioavailability of RES since its clearance is fast and it disappears from blood in minutes [23,24]. At the same time, there is a notable controversy about the potential biological activity of the metabolites of RES (glucuronates or sulfates) [25,26], and there is no information regarding the effect of these metabolites in ocular pathologies to the best of our knowledge. From a medicinal chemistry point of view, RES analogs or derivatives could enhance RES bioavailability or efficacy. Thus, several natural derivatives of RES have been investigated. For example, piceatannol (Figure 1, compound **2**) and pinosylvin (Figure 1, compound **3**) were able to mediate protection against oxidative stress in human RPE cells [27,28]. Pterostilbene (Figure 1, compound **4**) proved to be a good protective agent that may delay early retinal alterations induced by hyperglycemia in a rabbit model of type 1 diabetes mellitus through activation of the PI3K/AKT/GSK3β/NRF2 pathway [29]. A methanol extract of *Dipterocarpus tuberculatus* containing ε-Viniferin (Figure 1, **5**), a RES dimer, together with other minor components such as asiatic acid, ellagic acid and gallic acid, showed protective effects in a blue light-caused macular degeneration in mice through improvement in the thickness of the whole retina, including the photoreceptor layer [30].

From a medicinal chemistry point of view, RES has been considered a hit compound that can be further optimized to a lead compound. Several strategies have been carried out such as the design and synthesis of RES analogs, derivatives, hybrids and prodrugs [31,32,33,34,35,36]. Interestingly, Crauste et al. have developed RES lipophenols as retinoprotective agents and have shown these compounds were able to reduce oxidative damage in RPE cells [37,38].

**Figure 2:**
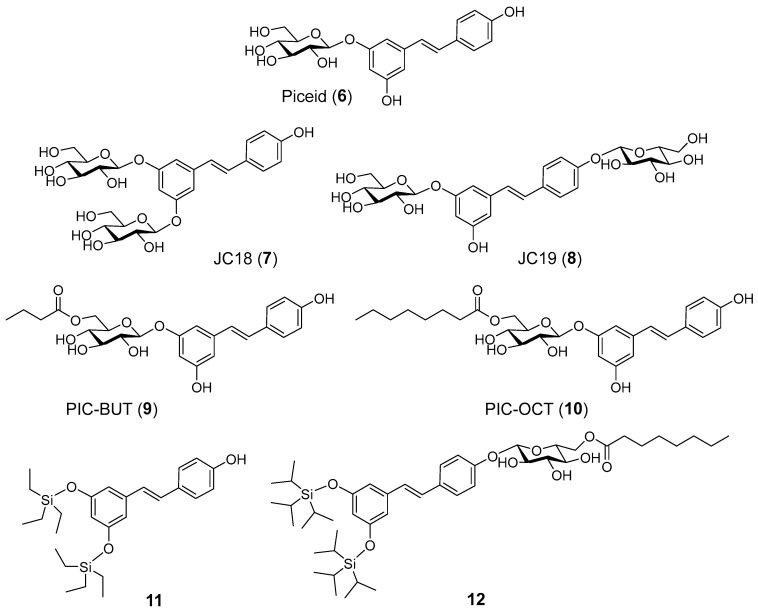
RES derivatives and prodrugs examined in this work.

Our research group has reported acyl- and glucosyl- RES derivatives as prodrugs and examined them as anti-inflammatory [39] and anticancer drugs [40]. Later, we focused on the preparation of alkyl- and silyl- RES derivatives and their prodrugs as potential treatments of neurodegenerative diseases [41,42]. In the field of ophthalmic diseases, we found that a RES prodrug, 3,4′-di-β-glucosyl resveratrol (JC19) (Figure 2, compound **8**), showed a retinoprotective effect in retinal degeneration 10 (rd10) mice, an experimental mouse model of autosomal recessive retinitis pigmentosa (RP) [43]. Treatment with JC19 via subretinal (SR) injection delayed the loss of rod photoreceptor in the rd10 mouse model, maintaining the expression of rhodopsin and preserving their electrical responses to light stimuli.

Retinitis pigmentosa (RP) represents a group of inherited retinal disorders marked by the gradual deterioration of photoreceptors. This degeneration initially impacts rod cells, which are responsible for low-light vision, followed by the subsequent degeneration of cone cells. Globally, RP affects about 1 in 4000 people [44]. The condition often begins with night blindness and a narrowing of peripheral vision, gradually advancing toward central vision loss and, in advanced stages, potentially leading to complete blindness. The pathophysiology of RP involves genetic mutations leading to the dysfunction and death of photoreceptors and the subsequent degeneration of the RPE. Currently, there is no cure for RP, and treatment options are limited. However, emerging therapies, such as gene therapy, retinal implants, and pharmacological interventions, aim to slow disease progression or restore some level of vision in affected individuals.

**Figure 3:**
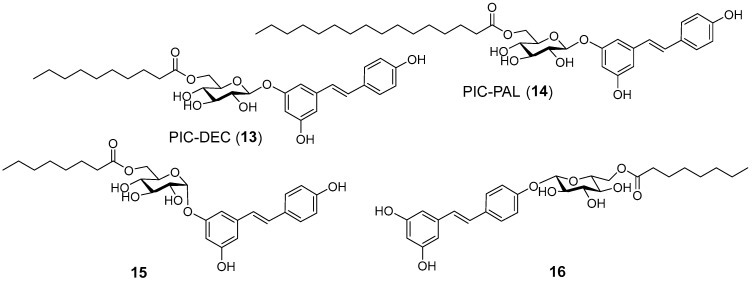
PIC-OCT derivatives prepared in this work.

In this work, we have investigated the potential as retinoprotective agents of a series of RES derivatives that have previously shown neuroprotective activity in a zebra fish model that included glucosyl-, acylated glucosyl-, alkyl- and silyl- RES derivatives (Figure 2) [41,42]. Then, we continued the study with the design, synthesis and evaluation of a second family of compounds (Figure 3) around piceid octanoate (PIC-OCT), the most efficient molecule of the first series (Figure 2, compound **10**).

## 2. Results and Discussion

### 2.1. Chemistry

Piceid (PIC), resveratrol 3-β-glucoside (Figure 2, compound **6**), is commercially available. 3,5-Di-β-glucosyl resveratrol (JC18) (Figure 2, compound **7**) was prepared from RES by random silyl protection, followed by glycosylation with peracetylated glucose trichloroacetimidate and boron trifluoride etherate, and final acetyl deprotection with NaOMe in methanol as reported previously [39]. PIC acylated compounds **9** and **10** (Figure 2) were synthesized by enzymatic acylation with Novozym435 (immobilized lipase from *Candida antarctica B*) and vinyl butyrate or vinyl octanoate, respectively, in *tert*-butanol as reported [39]. Random silylation of RES with the corresponding triethylsilyl chloride and triethylamine in dichloromethane (DCM) resulted in a mixture of trisilyl-, disilyl- and monosilyl- RES derivatives that was purified by flash column chromatography to isolate 3,5-ditriethylsilyl RES derivative **11** (Figure 2). The synthesis of compound **12** (Figure 2) was carried out by glycosylation of 3,5-ditriisopropyl silyl resveratrol, obtained by random silylation and column purification, followed by acetyl deprotection and final enzymatic acylation as previously described [42].

**Scheme 1:**
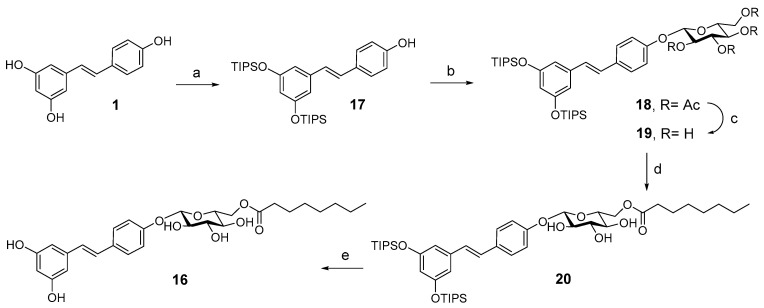
Synthesis of PIC-OCT regioisomer **16** from RES. (a) 1. Vinyl acetate, Novozym435^®^, 2-methylbutanol, 65 °C, overnight; 2. TIPSꞏOTf, DIPEA, DCM/THF; 3. NaOMe, MeOH/DCM; (b) 2,3,4,6-tetra-*O*-acetyl-α-D-glucopyranosyl trichloroacetimidate, BF_3_ꞏOEt_2_, anh. DCM; (c) NaOMe, MeOH; (d) Novozym435^®^, vinyl octanoate, t-BuOH, 65 °C, overnight; (e) HFꞏNEt_3_, THF.

A second family of RES derivatives was prepared trying to understand the relative relevance of the acyl chain and the glucose moiety on the efficacy of compound **10** (PIC-OCT) in delaying retinal degeneration in rd10 mice (Figure 3). PIC acylated compounds (**13** and **14**) were synthesized by enzymatic acylation as described [39]. Compound **15** (Figure 3) was prepared by enzymatic acylation of the corresponding resveratrol 3-α-glucoside with vinyl octanoate. Resveratrol 3-α-glucoside was synthesized enzymatically from RES using variant R134A of the sucrose phosphorylase from *Thermoanaerobacterium thermosaccharolyticum* (TtSPP) as reported previously [45]. This mutated enzyme has also been used to glucosylate other phenolic substrates such as phloretin [46]. Finally, compound **16** (Figure 3) was prepared in five steps from RES (Scheme 1). 3,5-Ditriisopropyl silyl resveratrol **17** was prepared from RES by enzymatic 4′ acetylation, followed by silylation and final deacetylation as previously reported [47]. Glycosylation of the disilyl RES derivative with 2,3,4,6-tetra-*O*-acetyl-α-D-glucopyranosyl trichloroacetimidate and boron trifluoride etherate resulted in compound **18**. Next, acetyl deprotection with NaOMe in methanol yielded **19** that was acylated with Novozym435 lipase using vinyl octanoate as the acyl donor and *tert*-butanol as solvent to obtain compound **20**. PIC-OCT regioisomer **16** was finally obtained after silyl deprotection of **20** with HFꞏNEt_3_ in THF.

### 2.2. Biology

#### 2.2.1. RES Derivative PIC-OCT (10) Preserves Efficiently Photoreceptors in rd10 Mice

**Figure 4:**
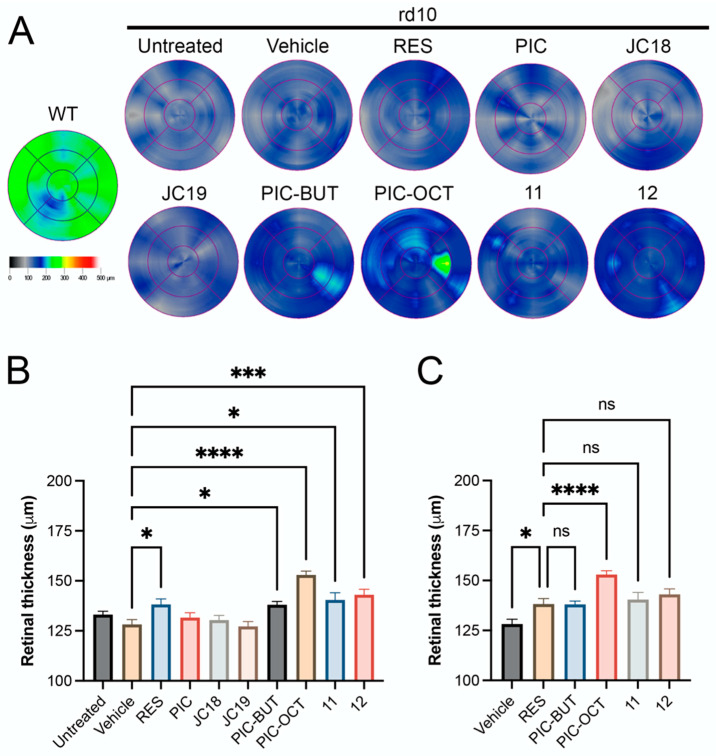
Retinal thickness of rd10 mice treated with different resveratrol (RES) derivatives. Retinal maps obtained by optical coherence tomography (OCT) scans of a wild type (WT) mouse (C57BL/6J) and untreated rd10 mice or treated with 5% DMSO (vehicle), or 5 mM RES, piceid (PIC), JC18, JC19, piceid butyrate (PIC-BUT), piceid octanoate (PIC-OCT), and compounds **11** and **12** (**A**). A representative image of each group is shown. The colorimetric scale represents the retinal thickness in µm. Six radial scans were measured to construct the retinal maps. The results are expressed as mean ± SEM. Nine measurements of retinal thickness were taken for each eye. The normality of the samples was evaluated by the Shapiro–Wilk test. The parametric one-way ANOVA test followed by Dunnett’s multiple comparisons test evaluated statistically significant differences between vehicle and each of the other groups (**B**) or between RES and vehicle, PIC-BUT, PIC-OCT, **11** and **12** (**C**). A *p*-value less than 0.05 was considered statistically significant. * *p* < 0.05; *** *p* < 0.001; **** *p* < 0.0001; (ns) not significant.

**Figure 5:**
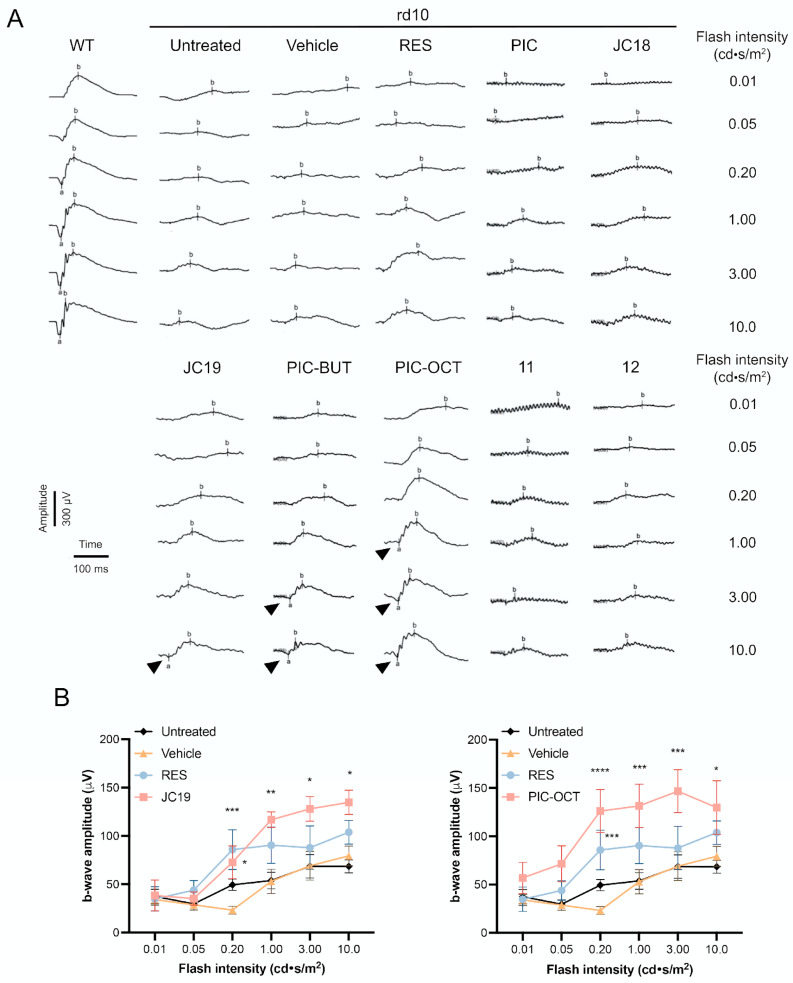
Representative ERG’s traces under dark-adapted conditions (**A**) and ERG quantification of b-wave amplitude of rd10 mice (**B**). The scotopic vision was evaluated in wild type (WT) and rd10 mice untreated or treated with 5% DMSO (vehicle) or with the different compounds (RES, PIC, JC18, JC19, PIC-BUT, PIC-OCT, **11** or **12**). The amplitude (µV) and time (ms) scales are shown. Different increasing flash intensities were tested (cd·s/m^2^). The b-wave (b) and the a-wave (a; arrowheads) are shown (**A**). The graphs represent the quantification of b-wave amplitude in untreated, vehicle, RES-, JC19- and PIC-OCT-treated groups (**B**). The results of JC19 and PIC-OCT groups are shown in two different graphs (**B**). The parametric two-way ANOVA test followed by Dunnett’s multiple comparisons test evaluated statistically significant differences between vehicle and each of the other groups. A *p*-value less than 0.05 was considered statistically significant. * *p* < 0.05; ** *p* < 0.01; *** *p* < 0.001; **** *p* < 0.0001.

**Figure 6:**
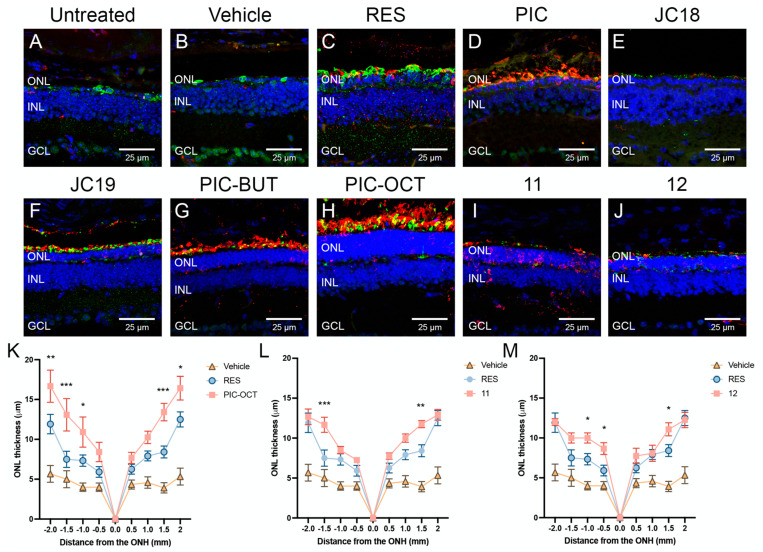
Histological evaluation of rd10 mouse retinas. Immunostaining (**A**–**J**) and quantification of outer nuclear layer (ONL) thickness (**K**–**M**) of untreated rd10 mouse retinas, or treated with vehicle or with the different compounds (RES, PIC, JC18, JC19, PIC-BUT, PIC-OCT, **11** or **12**) 15 days after subretinal injections are shown. The retinal sections were immunostained with anti-rhodopsin (**A–J**; red), and anti-opsin (**A–J**; green) antibodies to evaluate the expression of these rod and cone markers, respectively (**A**–**J**). DAPI dye (**A–J**; blue) was used to stain the nuclei of retinal layers. Scale bars represent 25 µm. ONL: outer nuclear layer; INL: inner nuclear layer; GCL: ganglion cell layer. The graphs represent the mean ± SEM of ONL thickness in sagittal retinal sections of untreated or treated rd10 mice (**K**–**M**). The ONL thickness were measured at −2, −1.5, −1, −0.5, 0, 0.5, 1, 1.5, and 2 mm from the optic nerve head (ONH). The parametric two-way ANOVA test followed by Dunnett’s multiple comparisons test evaluated statistically significant differences between RES and each of the other groups. A *p*-value less than 0.05 was considered statistically significant. * *p* < 0.05; ** *p* < 0.01; *** *p* < 0.001.

The rd10 mouse is a well-characterized autosomal recessive RP model. These mice carry a spontaneous missense mutation in exon 13 of the beta subunit of the rod phosphodiesterase gene (*pde6b*) [48]. In this model, degeneration of the rod photoreceptors begins on postnatal day 16 (P16), histology at three weeks of age (P21) shows retinal degeneration, with maximum cell death at P25, and is completed within one month (P30) [49]. The degeneration starts in the rods and continues with cones, which at P60 are atrophic, and the retina is completely remodeled. The RPE cells are also affected in later stages (P45) [50]. The degeneration in this model occurs due to the loss of function of the beta subunit of the protein complex pde6, which ultimately leads to a continuous entry of Ca^++^ in the rods with significant production of reactive oxygen species (ROS), infiltration of macrophages and activation of cell death mechanisms such as parthanatos through the dysregulation of poly(ADP-ribose) polymerase-1 (PARP1) expression [51]. Rd10 mice were injected into the SR space at P14 with 1 µL of vehicle (5% DMSO in PBS) or 5 mM solution of different compounds: RES, PIC (**6**), JC18 (**7**), JC19 (**8**), PIC-BUT (**9**), PIC-OCT (**10**), **11** and **12**. Fourteen days after injection (P28), optical coherence tomography (OCT) (Figure 4) and electroretinogram (ERG) studies (Figure 5, Supplementary Figures S1 and S2) were performed to determine retinal thickness and electrical responses of retinal cells, respectively. Once OCT and ERG were recorded, the treated animals were euthanized, and the eyes were quickly excised and processed for histological evaluation and the immunostaining of photoreceptor markers (Figure 6).

The in vivo quantification of the total retina (TR) thickness in the rd10 mice treated with the different molecules was carried out through OCT scans as described in the experimental section (Figure 4). The retinal map, which displays the thickness of the retina represented through a colorimetric scale, is shown in Figure 4A. The thickest retinas were observed in the groups treated with the compounds RES (mean 138.2 ± SEM 2.8 µm), PIC-BUT (138.0 ± 1.7 µm), PIC-OCT (153.0 ± 1.9 µm), and the compounds **11** (140.4 ± 3.6 µm) and **12** (143.0 ± 2.7 µm) (Figure 4A,B). PIC-OCT-treated mice showed the thickest retinas in rd10 mice, although thinner than wild type (WT) mice (Figure 4A). Nevertheless, the differences in the retinal thickness were statistically significant in the mice treated with the compounds RES, PIC-BUT, PIC-OCT, **11** and **12** when comparing them with the SR vehicle-treated group (128.2 ± 2.4 µm) (Figure 4B). The only compound that shows statistically significant differences with RES retinal thickness (138.2 ± 2.8 µm) was PIC-OCT (Figure 4C). No statistical differences were observed in retinal thickness between the untreated and vehicle-treated group (Figure 4B).

OCT is a non-invasive test to take cross-section images of the retina using light waves that allow segmenting and evaluating the thickness of the different retinal layers. In this study, we measure the TR thickness, which is defined as the distance between the internal limiting membrane (ILM) and the second highly reflective interface (RPE/Choroids complex) of the outer highly reflective bands (OHRBs). The TR thickness of WT mice at P28 was around 220 µm [49]. The mean TR thickness in the rd10 SR injected with vehicle was 128.2 µm, representing a 41.7% reduction in retinal thickness compared to a normal mouse retina. The TR thickness of SR PIC-OCT-treated mice at the same age was 153.0 µm, representing just a 30.5% reduction in retinal thickness (Figure 4A). Therefore, a single SR injection of 5 mM PIC-OCT preserved the TR thickness of rd10 mice at P28.

The ERG, which measures the retina’s electrical response to visual stimuli, is markedly affected in rd10 mice, showing a progressive decline in the a- and b-wave amplitude, which are extinguished after three weeks of age [52]. Fourteen days after injection (P28), ERG studies were performed to determine the electrical responses of retinal cells under dark-adapted conditions to evaluate scotopic vision (Figure 5 and Supplementary Figure S1) and light-adapted conditions to evaluate photopic vision (Supplementary Figure S2). For scotopic ERG recording, different flash intensities divided into six steps of 0.01, 0.05, 0.2, 1, 3 and 10 candelas (cd)·s/m^2^ were used. In dark-adapted conditions, the ERG waveform has two components, an early negative a-wave originated for photoreceptors cells and a positive b-wave. In electroretinography, following dark adaptation, a dim flash below the cone response threshold is used to obtain the dark-adapted 0.01 cd·s/m^2^ ERG. In this test, the b-wave component originates primarily from rod-driven bipolar cells, depending on the functional state of the rod photoreceptors. At higher flash intensities, such as 3 or 10 cd·s/m^2^, the response involves rod and cone systems, with the rod system typically dominant in the resulting signal [53]. According to the current International Society for Clinical Electrophysiology of Vision (ISCEV, 2022) standard, the b-wave is driven primarily by the activity of rod-driven On-bipolar cells. Additionally, a slower, prolonged positive component is observed. The source of this slower and prolonged positive component remains under investigation, but it has been suggested that both bipolar cells and Müller glial cells play a role [54]. Similarly, in light-adapted ERG conditions, such as the 3 cd·s/m^2^ protocol, the b-wave results from a combination of On- and Off-bipolar cell activity mediated by L-, M- and S-cone contributions [53]. The amplitude of this photopic b-wave increases by increasing the flash intensity, which was 3, 5, 10, 15, 20 and 30 cd·s/m^2^.

PIC-OCT-injected mice showed the highest b-wave amplitudes when compared with untreated or the other treated groups under dark-adapted conditions (Figure 5A,B and Supplementary Figure S1). At the highest flash intensities, the negative a-wave was observed in the JC19-, PIC-BUT- and PIC-OCT-treated groups (Figure 5A; arrowheads). When evaluating the quantification of b-wave amplitude of each compound separately, isolated significant differences were observed in some intensities in the groups treated with RES, PIC, JC19, PIC-OCT and the compounds **11** and **12**. However, the highest amplitudes that were consistently maintained at various intensities were only observed in the groups treated with JC19 and PIC-OCT (Figure 5B and Supplementary Figure S1). Under light-adapted conditions, only JC19-treated mice showed a consistent increase in b-wave amplitude, and PIC-BUT and the compounds **11** and **12** showed statistically significant differences at the higher intensities (Supplementary Figure S2).

Retinal sections of untreated and vehicle-treated rd10 mice show massive loss of nuclei at the outer nuclear layer (ONL) of the retina (the layer in which photoreceptor nuclei are located), which is accompanied by loss of the specific markers of rods (rhodopsin; Figure 6A–J, red) and cones (opsin; Figure 6A–J, green). PIC-OCT-treated mice showed the thickest ONL and preservation of rod and cone markers (Figure 6H). The quantification of ONL thickness in WT mouse retinas varies according to the mouse strain, age or technique used to measure it. The values of ONL thickness can fluctuate when it is measured directly in retinal sagittal sections or by spectral OCT, respectively. In rd10 mice, a significant atrophy of the ONL occurs in a central to peripheral gradient as is shown in Figure 6K. RES showed thicker retinas than the vehicle-treated group (Figure 6K). Nevertheless, statistical differences in ONL thickness were only observed in PIC-OCT (Figure 6K) and compounds **11** (Figure 6L) and **12** (Figure 6M) when compared with the RES-treated group. The ONLs in the rest of evaluated compounds were similar to RES (Supplementary Figures S3 and S4). PIC-OCT-treated mice showed the thickest ONL (Figure 6K).

The results of this initial screening study demonstrate that the RES derivative, PIC-OCT, is highly effective in preserving photoreceptors in rd10 mice, a well-established model of RP. Following a single SR injection of PIC-OCT, significant preservation of total retinal thickness was observed (Figure 4), along with improved electrical responses, as measured by ERG under scotopic conditions (Figure 5). Among the compounds tested, PIC-OCT exhibited the highest b-wave amplitudes, indicating superior rod photoreceptor function. Immunohistochemical analysis further revealed enhanced preservation of the ONL, where photoreceptors are located, as well as the retention of rod and cone markers (Figure 6). These findings from this initial screening highlight the strong potential of PIC-OCT as a RES derivative for the treatment of RP, effectively mitigating photoreceptor damage and improving retinal function.

#### 2.2.2. RES Derivative PIC-OCT (10) Shows Dose–Response Activity in rd10 Mice

**Figure 7:**
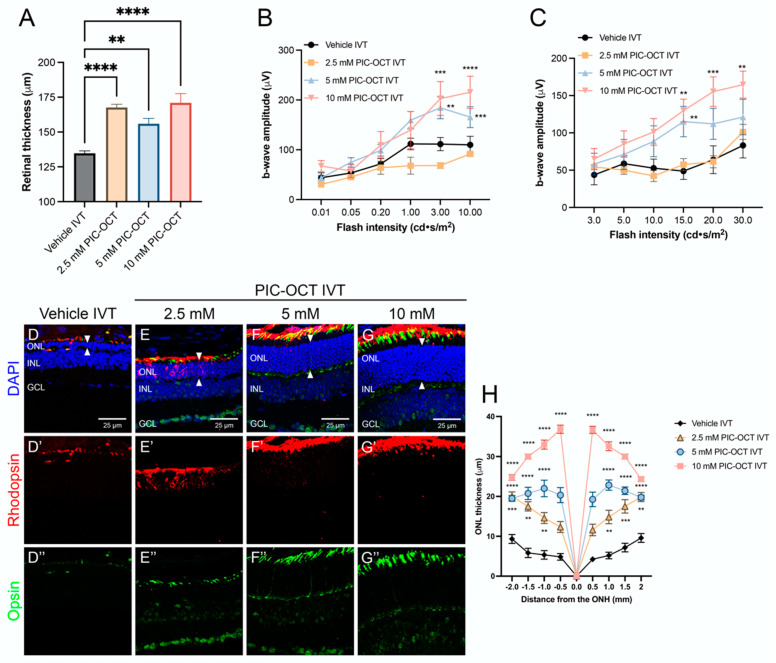
Effect of treatment with different doses of PIC-OCT on the rd10 mouse retinas. Quantification of retinal thickness by optical coherence tomography (OCT) (**A**). Electroretinogram (ERG) quantification of b-wave amplitude in dark-adapted conditions (**B**) and light-adapted conditions (**C**). Immunostaining (**D**–**G”**) and quantification of outer nuclear layer (ONL) thickness (**H**) of rd10 mice treated with vehicle or intravitreal (IVT) injections of 2.5, 5 and 10 mM PIC-OCT. The retinal sections were immunostained with anti-rhodopsin (red; **D’**–**G’**) and anti-opsin (green; **D”**–**G”**) antibodies to evaluate the expression of these rod and cone markers, respectively (**D**–**G”**). DAPI dye (blue; **D**–**G**) was used to stain the nuclei of retinal layers. Arrowheads indicate the ONL. Scale bars represent 25 µm. ONL: outer nuclear layer; INL: inner nuclear layer; GCL: ganglion cell layer. The graphs represent the mean ± SEM of retinal thickness (**A**), b-wave amplitude (**B**,**C**) and ONL thickness in sagittal retinal sections of treated rd10 mice (**H**). The ONL thickness were measured at −2, −1.5, −1, −0.5, 0, 0.5, 1, 1.5 and 2 mm from the optic nerve head (ONH). The parametric one-way ANOVA (**A**) or two-way ANOVA (**B**,**C**,**H**) test followed by Dunnett’s multiple comparisons test evaluated statistically significant differences between vehicle and each of the other groups. A *p*-value less than 0.05 was considered statistically significant. ** *p* < 0.01; *** *p* < 0.001; **** *p* < 0.0001.

In addition to the previously described effects of PIC-OCT on photoreceptor preservation, further experiments were conducted to evaluate the dose-dependent response of this compound in rd10 mice. These studies revealed a clear dose–response relationship, confirming the efficacy of PIC-OCT in preserving retinal structure and function in this RP model (Figure 7, Supplementary Figure S5). We decided to administer PIC-OCT via intravitreal (IVT) injection rather than the SR route used in earlier studies. The IVT route was chosen because it is easier to administer and can be routinely performed in ophthalmology clinics, making it a more practical option for potential clinical applications. Quantification of retinal thickness by OCT scans demonstrated a significant preservation of retinal thickness across all doses of PIC-OCT tested (2.5 mM, 5 mM, and 10 mM) when compared to vehicle-treated controls (Figure 7A, Supplementary Figure S5A). Although the 5 mM PIC-OCT dose appears thinner than the 2.5 mM dose in the cross-sectional images (Figure 7A), the difference between 2.5 and 5 mM groups is not statistically significant when comparing the average thickness data across doses. It is important to note that spontaneous retinal detachments have been reported in the rd10 model, which could have affected the thickness in the 2.5 mM group. It has been hypothesized that spontaneous retinal detachment in rd10 mice results from decreased pumping activity exerted by the RPE [49]. This phenomenon may contribute to the observed variability in retinal thickness, in addition to potential individual tissue responses to different PIC-OCT concentrations.

ERG recordings further supported the dose-dependent activity of PIC-OCT. In dark-adapted conditions, the b-wave amplitudes were significantly higher in PIC-OCT-treated mice compared to vehicle controls, with the 10 mM dose showing the most pronounced effect (Figure 7B, Supplementary Figure S5B). Under light-adapted conditions, a similar trend was observed, with increased b-wave amplitudes correlating with higher doses of PIC-OCT (Figure 7C, Supplementary Figure S5C). These findings indicate that PIC-OCT not only preserves retinal structure but also maintains the functional integrity of photoreceptors in a dose-dependent manner.

Immunostaining for rod and cone markers (rhodopsin and opsin) (Figure 7D–G) and subsequent quantification of the ONL thickness (Figure 7H) provided further evidence of the dose-dependent protective effects of PIC-OCT. Histological sections showed a marked preservation of the ONL in rd10 mice (Figure 7D–G; arrowheads) treated with higher doses of PIC-OCT, with the 10 mM dose resulting in the thickest ONL (Figure 7G). The ONL thickness measurements, taken at multiple points along the retinal axis, consistently demonstrated that higher doses of PIC-OCT led to greater preservation of photoreceptor nuclei, with the most significant effects observed at 10 mM (Figure 7H).

Notably, the highest dose of 10 mM PIC-OCT resulted in the greatest preservation of retinal thickness and expression of rhodopsin (Figure 7D’–G’) and opsin (Figure 7D”–G”). This dose-dependent effect suggests that increasing concentrations of PIC-OCT provide enhanced protection against photoreceptor degeneration in rd10 mice. These results collectively highlight the potential of PIC-OCT as a therapeutic agent for RP, with a clear dose–response relationship indicating that higher concentrations of the compound may provide greater neuroprotective benefits. The choice of IVT administration, due to its easy and routine use in clinical practice, further supports the feasibility of PIC-OCT as a treatment option in ophthalmological settings.

An opsin signal in the GCL and INL layers observed in Figure 7E”–G” may be attributed to the non-specific binding of the primary antibody to melanopsin, which can be expressed in specific subtypes of retinal ganglion cells. Although opsins are typically associated with photoreceptors, certain ganglion cell types in the retina express melanopsin, an opsin involved in non-visual functions such as circadian rhythm regulation and the pupillary light reflex. A key study in this area is Berson et al. (2002) [55], which identified intrinsically photosensitive retinal ganglion cells in the mammalian retina that express melanopsin. While it is uncommon to observe other types of opsins, such as cone opsins, in these layers, some studies suggest that changes in opsin expression or localization outside of photoreceptors can occur under conditions of cellular stress or degenerative alterations.

Interestingly, in a recent study, we observed that rd10 mice treated with PIC-OCT exhibited reduced photophobia, which we suggested might be due to improvements in the inner retina [56]. This raises the possibility that the persistence of melanopsin in these retinal layers could enhance the pupillary reflex in rd10 mice, potentially helping them adapt more effectively to changing light conditions and reduce their photophobic response. Photophobia is a common symptom in RP that can significantly impact quality of life. Therefore, based on these observations, PIC-OCT would prevent neurodegeneration of photoreceptors and help prevent symptoms associated with RP, such as photophobia.

Table 1 shows the summary of the values obtained in the quantification of retinal thickness measured by OCT and ERG in WT or rd10 mice treated with IVT injections of vehicle or 10 mM PIC-OCT. The data in Table 1 indicate that the treatment’s most significant effects were observed in functional ERG tests rather than in retinal thickness preservation. Within these functional tests, the improvement in cone response (light-adapted ERG) was notably greater than in rod response (dark-adapted ERG). Specifically, the light-adapted ERG response showed a 41.9% improvement with PIC-OCT treatment over the vehicle, compared to a 22.4% improvement in the dark-adapted ERG. Conversely, the OCT measurement showed a relatively modest improvement in retinal thickness preservation, with a 16.5% increase over the vehicle. This highlights the treatment’s more pronounced impact on retinal function, especially in cone-mediated vision.

#### 2.2.3. Acyl Chain Length, Carbohydrate Position and Configuration Are Key for Delaying Retinal Degeneration by PIC-OCT (10) in rd10 Mice

**Figure 8:**
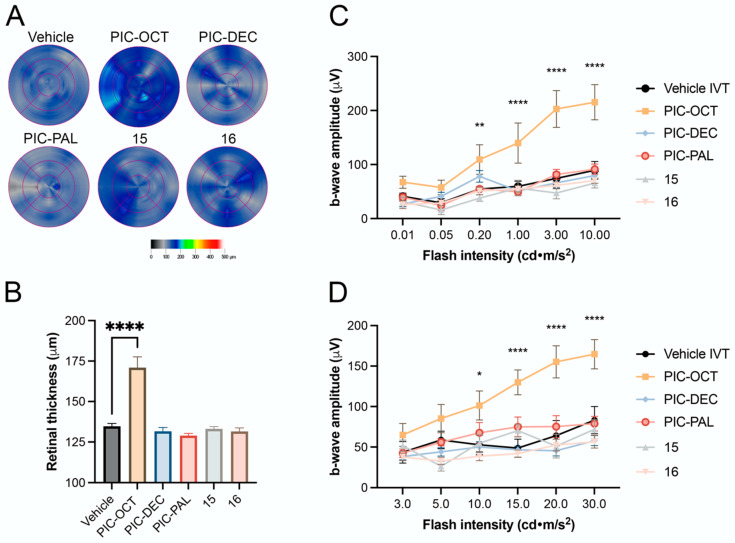
Effect of PIC-OCT and different acyl chain length and carbohydrate position on the rd10 mouse retinas. Retinal maps obtained by optical coherence tomography (OCT) scans of rd10 mice or treated with 5% DMSO (vehicle), or piceid octanoate (PIC-OCT), and compounds PIC-DEC, PIC-PAL **15** and **16** (**A**). Representative images of each group are shown. The colorimetric scale represents the retinal thickness in µm. Six radial scans were measured to construct the retinal maps. The graphs represent the mean ± SEM of retinal thickness (**B**), b-wave amplitude in dark-adapted (**C**) and light-adapted conditions (**D**). The parametric one-way ANOVA (**B**) or two-way ANOVA (**C**,**D**) test followed by Dunnett’s multiple comparisons test evaluated statistically significant differences between the vehicle and each of the other groups. A *p*-value less than 0.05 was considered statistically significant. * *p* < 0.05; ** *p* < 0.01; **** *p* < 0.0001.

The effects of PIC-OCT and related derivatives with different acyl chain lengths and carbohydrate positions on the retinas of rd10 mice are shown in Figure 8. Retinal maps obtained by OCT scans (Figure 8A) show variability in retinal thickness among the treated groups. Notably, treatment with PIC-OCT resulted in significant preservation of retinal thickness compared to the vehicle (5% DMSO). Quantitative analyses of retinal thickness (Figure 8B) revealed that PIC-OCT maintained retinal thickness and ONL integrity more effectively than any of the other compounds tested. Additionally, the b-wave amplitude results under dark-adapted (Figure 8C, Supplementary Figure S6A) and light-adapted conditions (Figure 8D, Supplementary Figure S6B) showed that PIC-OCT significantly improved retinal function compared to the other treatments.

Despite modifications in the carbon chain length and carbohydrate position among the different molecules tested, only PIC-OCT demonstrated a therapeutic effect by preserving retinal thickness and visual function in the rd10 mouse model. The lack of efficacy on those derivatives with different chain lengths could be attributed to the role of octanoate itself if it becomes liberated into the photoreceptors and can exert a potential anti-inflammatory effect or a neuroprotective effect on the photoreceptor cells by restoring cell metabolism. However, PIC-OCT analogs with the same “components” (resveratrol + glucose + octanoate) but different spatial disposition (sugar location or configuration) do not show any efficacy on delaying retinal degeneration. These results point to the fact that the entire structure of PIC-OCT is needed for displaying the observed biological effect. In fact, when rd10 mice were treated with a combination of RES and octanoic acid, no therapeutic effect was observed. The superiority of PIC-OCT may be attributed to better penetration, stability, or interaction with retinal tissues or specific targets, which allows for greater preservation of retinal structure and function under degenerative conditions. It is important to remark that PIC-OCT degrades into resveratrol sulfate metabolites after several hours in cell culture, at least in MCF7, HT-29 and Hek-293 cells [40]. In animal models, biodistribution studies of PIC-OCT are in progress to assess this relevant point. Overall, these findings underscore the unique therapeutic potential of PIC-OCT compared to other acylated piceid variants.

#### 2.2.4. PIC-OCT Reduces Microglial Migration and PARP1 Expression in the rd10 Retina

Given the anti-inflammatory properties of the PIC-OCT, we sought to investigate its effect on retinal inflammation in the rd10 mouse model. Apart from being characterized by progressive photoreceptor cell death, this model exhibited microglia activation and heightened gliosis, making it an ideal system for testing anti-inflammatory therapies. One key feature of retinal inflammation is the activation of Müller cells and microglia, two glial cells that respond to retinal damage. Müller cells express glial fibrillary acidic protein (GFAP), while microglia can be marked by ionized calcium-binding adaptor molecule 1 (Iba-1).

**Figure 9:**
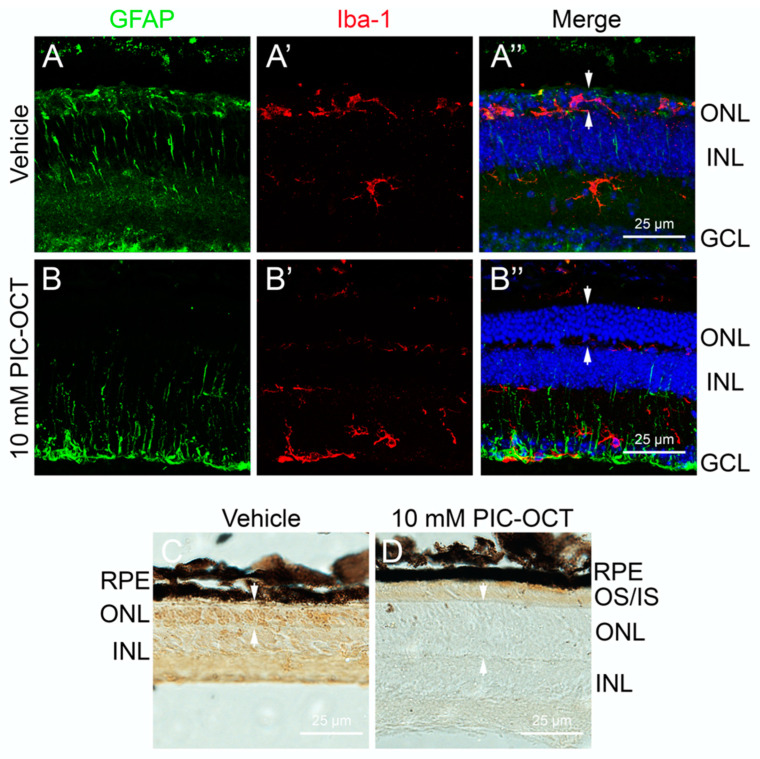
Effect of PIC-OCT on microglial migration and poly (ADP-ribose) polymerase-1 (PARP1) expression. Rd10 mice were intravitreally (IVT) injected with 10 mM PIC-OCT or vehicle. At P28, the mice were sacrificed, and retinal sections were immunostained with anti-glial fibrillary acidic protein (GFAP) (**A**,**B**; green), anti-ionized calcium-binding adaptor molecule 1 (Iba-1) (**A’**,**B’**; red), and DAPI (**A”**,**B”**; blue). Additionally, other retinal sections were prepared for immunocytochemistry using PARP1 and detected with diaminobenzidine (DAB) (**C**,**D**). RPE: retinal pigment epithelium; IS/OS: inner segment/outer segment; ONL: outer nuclear layer; INL: inner nuclear layer; GCL: ganglion cell layer. The arrows indicate the ONL thickness (**A”**,**B”**,**C**,**D)**. Scale bars represent 25 μm.

In our study, we observed a significant reduction in reactive gliosis in Müller cells due to PIC-OCT treatment. In PIC-OCT-treated rd10 mice, GFAP immunopositivity was limited to the ganglion cell layer, and the characteristic dendritic processes of reactive Müller cells were notably shorter (Figure 9B). In contrast, vehicle-treated rd10 mice showed extensive GFAP expression, with long, reactive processes extending through the ONL (Figure 9A).

Additionally, we examined microglial activation by analyzing Iba-1 expression. In untreated rd10 mice, we observed high levels of Iba-1 in the ONL, indicating robust microglial activation (Figure 9A’). However, Iba-1 expression was significantly reduced in PIC-OCT-treated mice and confined primarily to the ganglion cell layer (Figure 9B’). This reduction in microglial migration and reactivity further supports the anti-inflammatory role of PIC-OCT in the retina.

Finally, we assessed the expression of PARP1, a crucial enzyme involved in DNA repair, but its overactivation can lead to a form of programmed cell death known as parthanatos, which has been implicated in neurodegenerative conditions, including retinal degeneration. This form of cell death is distinct from apoptosis and necrosis and involves the release of poly ADP-ribose (PAR) polymers that lead to mitochondrial dysfunction. In retinal degeneration, PARP1 overactivation is a marker of pathological processes contributing to photoreceptor loss.

Vehicle rd10 mice exhibited strong PARP1 staining throughout the retina, particularly in the ONL, correlating with extensive cellular damage and photoreceptor death (Figure 9C). In contrast, PIC-OCT-treated rd10 mice showed no detectable PARP1 signal, indicating that the compound PIC-OCT not only reduces inflammation but also protects against parthanatos-driven cell death (Figure 9D). The retinas of PIC-OCT-treated mice appeared thicker and healthier, further underscoring the neuroprotective effects of the treatment. In a previously published paper by our group, we observed that PIC-OCT reduced the nuclear expression of PARP1 and prevented nuclear translocation of apoptosis-inducing factor in vitro. In addition, in rd10 mice, PIC-OCT treatment inhibited PAR polymer formation [56], supporting the hypothesis that the parthanatos pathway is inhibited by PIC-OCT treatment.

## 3. Conclusions

Herein are shown the results of RES derivatives, specifically PIC-OCT, as retinoprotective agents in the rd10 mouse model of RP. This study demonstrates that PIC-OCT significantly delays photoreceptor degeneration, which is evident through both functional assessments, such as the amplitude of the b-wave in ERG, and structural preservation, like the thickness of the retina and ONL. These findings highlight a robust neuroprotective effect, likely due to the compound’s ability to mitigate retinal cell degeneration.

Moreover, PIC-OCT emerged as the most effective molecule among those RES derivatives explored. The results clearly showed that PIC-OCT outperformed other closely related compounds, including PIC-DEC or PIC-PAL compounds, in the retina’s functional and structural preservation. Moreover, compounds **15** and **16**, isomers of PIC-OCT bearing an alpha glucosyl unit and containing the 6′-octanoyl β-glucosyl unit at a different OH in the stilbene structure, respectively, did not show any retinoprotective activity. Therefore, although **15** and **16** contain the same “units” as PIC-OCT, they are not active, indicating that the specific substitution of RES in PIC-OCT is needed as an entire molecule to display the therapeutic effect.

In conclusion, PIC-OCT could be a good leading candidate for the development of therapies for RP and potentially other neurodegenerative retinal diseases due to its superior efficacy in preserving retinal structure and function. Future directions in this work should lead to new ways of administering this type of molecule to the patients, such as eye drops, which are much less aggressive than the actual intravitreal injections used today for treatment of retinal diseases.

## 4. Materials and Methods

### 4.1. Chemistry

All solvents and chemicals were used as purchased without further purification. All reactions were monitored by TLC on precoated silica gel 60 plates F254 (Merck, Burlington, MA, USA) and detected by heating after staining with H_2_SO_4_:EtOH (1:9, *v*/*v*) or Mostain (500 mL of 10% H_2_SO_4_, 25 g of (NH_4_)_6_Mo_7_O_24_·4H_2_O, 1 g Ce(SO_4_)_2_·4H_2_O). Products were purified by flash chromatography with silica gel 60 (200–400 mesh). Eluents are indicated for each particular case. NMR spectra were recorded on Bruker Advance 300, 400 or 500 MHz [300, 400 or 500 MHz (^1^H), 75, 101 or 126 (^13^C)] NMR spectrometers, at room temperature for solutions in CDCl_3_ or CD_3_OD. Chemical shifts are referred to the solvent signal. Chemical shifts are in ppm. High-resolution mass spectra (HRMS) were obtained on an ESI/quadrupole mass spectrometer (WATERS, ACQUITY H CLASS).

#### 4.1.1. Preparation of Compound **13** (PIC-C10)

- 3-*O*-(6′-*O*-decanoyl)-β-D-glucopyranosyl resveratrol

To a mixture of PIC **6** (100–150 mg, 1 equiv.) and vinyl decanoate (20 equiv.) in 15–20 mL of *tert*-butyl alcohol, Novozym435 (Novozymes) (100–150 mg) was added. The mixture was stirred in an orbital shaker at 60 ˚C for 16 h. The enzyme was decanted and separated. The solvent was evaporated, and the product was purified by flash column chromatography (ethyl acetate/MeOH from 1:0 to 9:1) to yield compound **13** (79%). ^1^H NMR (400 MHz, Methanol-*d*_4_) δ 7.35 (t, *J* = 6.0 Hz, 2H), 7.06–6.97 (m, 1H), 6.80 (m, 4H), 6.67 (d, *J* = 5.0 Hz, 1H), 6.46 (d, *J* = 6.1 Hz, 1H), 4.94 (t, *J* = 5.6 Hz, 1H), 4.45 (d, *J* = 11.5 Hz, 1H), 4.26 (t, *J* = 6.2 Hz, 1H), 4.08 (dd, *J* = 9.5, 4.8 Hz, 1H), 3.72 (d, *J* = 7.9 Hz, 1H), 3.55 (d, *J* = 6.1 Hz, 2H), 2.27 (t, *J* = 7.3 Hz, 2H), 2.08–1.89 (m, 2H), 1.47 (t, *J* = 7.0 Hz, 2H), 1.16 (m, 10H), 0.89–0.82 (m, 3H); ^13^C NMR (101 MHz, MeOD) δ 174.32, 158.80, 158.15, 157.04, 139.93, 128.87, 128.65, 127.64, 125.43, 115.25, 107.19, 105.54, 103.04, 100.55, 76.50, 73.93, 73.44, 70.60, 63.52, 60.30, 47.81, 33.69, 31.70, 29.23, 29.08, 29.05, 28.77, 24.64, 22.40, 19.69, 13.27; TOF MS ES+ Calculated mass for C_30_H_41_O_9_ [M + H] = 545.2751, Found mass [M + H] = 545.2753.

#### 4.1.2. Preparation of Compound **15**

- 3-*O*-(6′-*O*-octanoyl)-α-D-glucopyranosyl resveratrol (**15**)

A mixture of resveratrol α-glucoside [43] (100–150 mg, 1 equiv.) and vinyl octanoate (20 equiv.) in 15–20 mL of *tert*-butyl alcohol was stirred in an orbital shaker at 60 ˚C for 16 h. The enzyme was decanted and separated. The solvent was evaporated, and the product was purified by flash column chromatography (ethyl acetate/MeOH from 1:0 to 9:1) to yield compound **15** (48%). ^1^H NMR (400 MHz, MeOD) δ 7.39 (d, *J* = 8.5 Hz, 2H), 7.03 (d, *J* = 16.3 Hz, 1H), 6.91–6.74 (m, 4H), 6.65 (t, *J* = 1.8 Hz, 1H), 6.51 (t, *J* = 2.1 Hz, 1H), 5.48 (d, *J* = 3.7 Hz, 1H), 4.45 (dd, *J* = 11.7, 2.0 Hz, 1H), 4.17 (dd, *J* = 11.8, 7.5 Hz, 1H), 3.94–3.80 (m, 1H), 3.60 (dd, *J* = 9.7, 3.6 Hz, 1H), 3.33 (dd, *J* = 3.4, 1.7 Hz, 2H), 2.21 (t, *J* = 7.6 Hz, 2H), 1.44 (dd, *J* = 9.4, 4.8 Hz, 2H), 1.31–1.15 (m, 9H), 0.87 (t, *J* = 7.1 Hz, 3H); ^13^C NMR (126 MHz, MeOD) δ 174.03, 158.27, 158.19, 157.16, 139.95, 128.82, 128.56, 127.51, 125.28, 115.13, 107.11, 105.62, 103.13, 97.32, 73.64, 71.90, 70.79, 70.63, 63.33, 39.02, 33.62, 31.47, 28.72, 28.68, 28.64, 24.53, 22.26, 13.03; TOF MS ES+ Calculated mass for C_28_H_36_O_9_ [M + H] = 516.2359, Found mass [M + H] = 545.2753.

#### 4.1.3. Preparation of Compound **16**

- 3,5-*O*,*O*-di-triisopropylsilyl-4′-*O*-[(2,3,4,6-*O*-tetraacetyl)-β-D-glucopyranosyl] resveratrol (**18**)

3,5-ditriisopropylsilyl-resveratrol **17** (4.01 g, 7.42 mmol) [47] and 2,3,4,6-tetra-*O*-acetyl-α-D-glucopyranosyl trichloroacetimidate (10.93 g, 22.26 mmol) were dissolved in anhydrous dichloromethane (25 mL) under argon atmosphere and BF_3_·OEt_2_ (920 μL, 7.42 mmol) was added to the reaction mixture. The reaction was quenched after 30 min by addition of NEt_3_ (4 mL), concentrated in vacuo and purified by flash column chromatography (Hexane:AcOEt 3:1) to achieve compound **18** as a yellowish powder (5.82 g, 90% yield). ^1^H NMR (400 MHz, CDCl3) δ 7.50–7.38 (m, 2H), 7.04–6.82 (m, 3H), 6.63 (d, *J* = 2.2 Hz, 2H), 6.54 (s, 1H), 6.34 (t, *J* = 2.2 Hz, 1H), 5.37–5.23 (m, 2H), 4.31 (dd, *J* = 12.3, 5.2 Hz, 1H), 4.22–4.07 (m, 3H), 3.93–3.84 (m, 1H), 2.13–2.01 (m, 12H), 1.26 (td, *J* = 7.1, 3.6 Hz, 6H), 1.12 (d, *J* = 7.3 Hz, 36H); ^13^C NMR (101 MHz, CDCl_3_) δ 170.65, 170.27, 169.47, 169.38, 163.75, 157.06, 156.30, 138.93, 132.76, 128.09, 127.71, 117.14, 111.27, 99.04, 77.09, 72.71, 72.04, 71.20, 70.33, 70.08, 69.75, 69.40, 68.30, 67.34, 61.96, 61.22, 60.41, 21.03, 20.71, 20.64, 20.60, 20.58, 20.52, 17.92, 14.18, 12.67; TOF MS ES+ Calculated mass for C_46_H_70_O_12_Si_2_ [M + H] = 871.4484, Found mass [M + H] = 871.4507.

- 3,5-*O*,*O*-di-triisopropylsilyl-4′-*O*-β-D-glucopyranosyl resveratrol (**19**)

Compound **18** (5.82 g, 6.69 mmol) was dissolved in a 1:1 mixture of MeOH/DCM (20 mL) and 0.3 eq of NaOMe was added to the solution at r.t. The reaction was stopped after 45 min, neutralized by addition of amberlite IR-120 (H^+^), filtered and evaporated to afford 4.47 g of the desired compound **19** (95% yield). ^1^H NMR (400 MHz, MeOD) δ 7.46 (d, *J* = 8.9 Hz, 2H), 7.09 (d, *J* = 8.8 Hz, 2H), 6.92 (d, *J* = 13.6 Hz, 2H), 6.64 (d, *J* = 2.2 Hz, 2H), 6.31 (t, *J* = 2.1 Hz, 1H), 4.98 (d, *J* = 7.2 Hz, 1H), 3.96–3.68 (m, 4H), 3.53 (d, *J* = 11.7 Hz, 2H), 1.31–1.17 (m, 6H), 1.11 (d, *J* = 7.3 Hz, 36H); ^13^C NMR (101 MHz, MeOD) δ 165.21, 164.78, 157.18, 156.96, 139.44, 131.67, 128.01, 127.55, 126.98, 116.66, 111.08, 106.44, 100.71, 92.48, 76.53, 76.37, 74.65, 74.61, 73.44, 72.99, 71.83, 71.58, 69.87, 69.17, 61.07, 60.69, 48.03, 17.41, 12.59; TOF MS ES+ Calculated mass for C_38_H_62_O_8_Si_2_ [M + H] = 703.4061, Found mass [M + H] = 703.4072.

- 3,5-*O*,*O*-di-triisopropylsilyl-4′-*O*-(6′-*O*-octanoyl)-β-D-glucopyranosyl resveratrol (**20**)

To a mixture of compound **19** (4.47 g, 6.36 mmol) and vinyl octanoate (9.85 mL, 8 equiv.) in 50 mL of *tert*-butyl alcohol, Novozym435^®^ (Novozymes) (4.6 g) was added. The mixture was stirred in an orbital shaker at 60 ˚C for 16 h. The enzyme was decanted and separated. The solvent was evaporated, and the product was purified by flash column chromatography (Hexane/ethyl acetate from 1:3 to 1:5) to yield compound **20** (2.32 g, 44%). ^1^H NMR (400 MHz, MeOD) δ 7.55–7.41 (m, 2H), 7.07 (d, *J* = 8.7 Hz, 2H), 6.95 (q, *J* = 16.3 Hz, 2H), 6.66 (d, *J* = 2.1 Hz, 2H), 6.33 (d, *J* = 1.7 Hz, 1H), 4.93 (d, *J* = 14.6 Hz, 1H), 4.44 (dd, *J* = 11.8, 2.2 Hz, 1H), 4.26 (dd, *J* = 11.8, 7.3 Hz, 1H), 3.68 (td, *J* = 7.5, 3.8 Hz, 1H), 3.57–3.46 (m, 2H), 3.37 (ddd, *J* = 9.3, 6.2, 2.9 Hz, 1H), 2.42–2.23 (m, 4H), 1.60 (td, *J* = 9.6, 8.1, 3.9 Hz, 4H), 1.31 (d, *J* = 8.5 Hz, 7H), 1.30–1.22 (m, 6H), 1.14 (d, *J* = 7.3 Hz, 36H); ^13^C NMR (101 MHz, MeOD) δ 176.33, 173.86, 157.22, 156.98, 141.02, 139.55, 131.63, 128.01, 127.35, 126.83, 116.67, 111.00, 110.39, 100.58, 96.43, 76.48, 74.01, 73.42, 70.51, 63.40, 50.61, 33.81, 33.63, 31.50, 28.90, 28.85, 28.77, 28.75, 27.22, 24.74, 24.70, 22.35, 22.30, 17.15, 13.20, 13.10, 12.57.; TOF MS ES+ Calculated mass for C_46_H_77_O_9_Si_2_ [M + H] = 829.5106, Found mass [M + H] = 829.5125. Purity > 95%, checked by HPLC.

- 4′-*O*-(6′-*O*-octanoyl)-β-D-glucopyranosyl resveratrol (**16**)

To a solution of compound **20** (2.27 g, 2.74 mmol) in 15 mL of anhydrous THF, Et_3_N·3HF (2.68 mL,16.43 mmol) was slowly added. The reaction was stirred at room temperature for 4 h until full consumption of the starting material. The solvent was evaporated, and the crude was re-suspended in EtOAc and extracted with water (3 × 50 mL) and brine (3 × 50 mL). The organic layer was then purified by flash column chromatography (Hexane/ethyl acetate from 1:4 to 1:5) to yield compound **16** (1.24 g, 87%) as a yellowish powder. ^1^H NMR (400 MHz, MeOD) δ 7.47 (dd, *J* = 8.8, 4.8 Hz, 2H), 7.07 (d, *J* = 8.4 Hz, 2H), 7.05–6.83 (m, 2H), 6.57–6.45 (m, 2H), 6.21 (t, *J* = 2.1 Hz, 1H), 5.01–4.91 (m, 1H), 4.44 (dd, *J* = 11.8, 2.3 Hz, 1H), 4.27 (dd, *J* = 11.7, 7.2 Hz, 1H), 3.76–3.59 (m, 1H), 3.55–3.46 (m, 2H), 3.43–3.35 (m, 1H), 2.40–2.25 (m, 4H), 1.69–1.53 (m, 4H), 1.38–1.22 (m, 7H); ^13^C NMR (101 MHz, MeOD) δ 174.02, 158.38, 157.15, 139.62, 131.99, 127.48, 127.36, 127.32, 127.23, 116.68, 108.63, 106.07, 104.66, 101.67, 100.69, 76.55, 74.08, 73.51, 70.49, 69.58, 63.34, 33.81, 31.56, 28.94, 24.77, 22.39, 17.13, 13.11; TOF MS ES+ Calculated mass for C_28_H_37_O_9_ [M + H] = 517. 2438, Found mass [M + H] = 517.2438. Purity > 95%, checked by HPLC.

### 4.2. Biology

#### 4.2.1. Animal Handling

For in vivo experiments, homozygous B6.CXB1-Pde6brd10/J mice (from The Jackson Laboratory), known as rd10, were used. These mice were kept under controlled temperature conditions with a 12 h light/dark cycle and ad libitum access to food and water. Male and female mice were obtained from different litters of the same breeding parents. The sample size (n) in each group varied from 6 to 10, depending on the number of pups per litter, with each litter used for a specific experiment. All procedures were conducted in compliance with the standards established by the Spanish Laboratory Animal Science Association (SECAL) and the Federation of European Laboratory Animal Science Associations (FELASA), as well as the European Union Council Directive 2010/63/EU and ARVO’s guidelines for the use of animals in ophthalmic and vision research. Animal handling and experimental procedures were approved and supervised by the CABIMER Animal Experimentation Committee in Seville, Spain, and the Directorate General for Agricultural and Livestock Production of the Andalusian Regional Government (10/02/2021/010). Every effort was made to minimize both the number of animals used and their suffering.

#### 4.2.2. Compound Administration

Surgical procedures were conducted under general anesthesia by intraperitoneal injection of a ketamine/xylazine solution at a dose of 80/12 mg/kg body weight (BW). Each eye was topically anesthetized with 0.1% tetracaine and 0.4% oxybuprocaine, and pupils were dilated with one drop each of 10% phenylephrine and 1% tropicamide. To access the subretinal or intravitreal space, a 32-gauge needle was used to gently open the choroid 1 mm posterior to the sclerocorneal limbus. A single injection was administered using a 10 µL syringe (Hamilton, Switzerland) and a 33-gauge needle attached to an ultra-micropump (World Precision Instruments, Sarasota, FL, USA), delivering 1 µL containing 2.5, 5, or 10 mM RES derivatives dissolved in 5% DMSO. Finally, a drop of antibiotic (0.3% ciprofloxacin) was applied to each eye, and the animals were kept on a 37 °C pad until fully recovered. Both eyes were injected, and mice were sacrificed by cervical dislocation two weeks after subretinal injections.

#### 4.2.3. Ocular Coherence Tomography (OCT)

OCTs were performed as described by Pensado et al. [57]. Following pupil dilation and sedation of the mice, retinal scans were obtained using a Stratus time-domain optical coherence tomography system (Carl Zeiss, Jena, Germany). The protocol consisted of a series of six equally spaced linear sweeps with a common central axis, adjusted to cover 3 mm of the central retina and with a focus set to +12 diopters. Quantitative analysis included measurements of retinal thickness and the distance between the inner limiting membrane adjacent to the ganglion cell layer and the band connecting the outer segments of photoreceptors with the RPE (OCT Stratus Software 4.0, Carl Zeiss, Jena, Germany). A colorimetric map was used to represent retinal thickness.

#### 4.2.4. Electroretinogram (ERG)

Full-field ERGs were performed as described by Valdes-Sanchez et al. [43] and recorded in a ColorDome Ganzfeld (Diagnosys LLC, Lowell, MA, USA). For scotopic vision assessment, mice were dark-adapted overnight and anesthetized with ketamine/xylazine (80/12 mg/kg BW). After pupil dilation and application of local anesthesia on the cornea, lubricant gel (1% methylcellulose) was used to bridge the gap between the electrodes and the cornea. The band-pass filter cutoff frequencies were set between 0.312 and 300 Hz. A single white-flash pulse (6500 K) was applied with stimulus intensity at six levels of 0.01, 0.05, 0.2, 1, 3, and 10 cd·s/m^2^. Fifteen responses were averaged at each level with a 15 s interstimulus interval. For photopic vision assessment, mice were light-adapted for 10 min with background illumination of 30 cd/m^2^, and stimulus intensities of 3, 5, 10, 15, 20 and 30 cd·s/m^2^ were used to obtain photopic responses.

#### 4.2.5. Histology and Outer Nuclear Layer (ONL) Thickness

Transverse sections of treated and untreated rd10 mice were stained with hematoxylin and eosin (H&E). The number of nuclei in the ONL was counted in sections from five parallel series, and ONL thickness was measured at distances of −2, −1.5, −1, −0.5, 0, 0.5, 1, 1.5 and 2 mm from the optic nerve head (ONH).

#### 4.2.6. Immunofluorescence and Immunocytochemistry Experiments

Animals were sacrificed on postnatal day 28 (P28), after which the eyes were promptly excised and processed. For immunofluorescence, the eyes were fixed overnight in 4% paraformaldehyde in phosphate-buffered saline (PBS) at 4 °C. Fixed eyes were then cryoprotected for 8 h at room temperature in 20% sucrose-PBS and further overnight at 4 °C in 30% sucrose-PBS, prior to cryostat sectioning. Serial sections, 18 μm thick, were washed in 0.2% Triton X-100 in PBS and blocked with 1% bovine serum albumin in PBS at room temperature for 1 h. Primary antibody incubation (anti-rhodopsin, Abcam, the Netherlands; anti-opsin L/M, Millipore, Germany; anti-GFAP, Sigma-Aldrich, Germany; Iba-1, Wako, Germany) was carried out overnight at 4 °C. Following washing, samples were incubated with secondary antibodies (AlexaFluor^®^ anti-rabbit 488, anti-mouse 633) at room temperature for 1 h. After three washes, sections were mounted with a Vectashield containing DAPI (Vector Laboratories, Inc. Newark, CA, USA). For immunohistochemistry, samples were incubated with anti-PARP1 (Abcam, the Netherlands) and biotinylated anti-rabbit IgG (Vector Laboratories, Inc. Newark, CA, USA) for 1 h at room temperature. Immunoreactive signals were visualized using an avidin–biotin–peroxidase complex in a Tris-HCl buffer with 0.02% 3,3′-diaminobenzidine (DAB) and 0.005% H_2_O_2_.

## Figures and Tables

**Table 1:** Summary of the values obtained in the main tests performed on WT and rd10 mice treated with vehicle or PIC-OCT.

Test	WT (Mean)	Vehicle IVT (Mean)	10 mM PIC-OCT (Mean)	WT vs. Vehicle Loss (%)	WT vs. PIC-OCT Loss (%)	PIC-OCT vs. Vehicle Improvement (%)
OCT (µm)	220.7	134.7	171.0	39.0	22.5	16.5
10 DA ERG (µV)	470.9	109.9	215.4	76.7	54.3	22.4
30 LA ERG (µV)	194.2	83.3	164.7	57.1	15.2	41.9

WT: wild type; IVT: intravitreal; PIC-OCT: piceid octanoate; OCT: ocular coherence tomography; DA: dark-adapted; LA: light-adapted; ERG: electroretinogram.

## Data Availability

The original contributions presented in the study are included in the article/Supplementary Materials. Further inquiries can be directed to the corresponding author.

## Supplementary Materials

The following supporting information can be downloaded at: https://www.mdpi.com/article/10.3390/ph17111482/s1, Supplementary Figures and copies of 1H, 13C NMR, and HRM spectra for all new compounds.
