# Supplementary material for: Synthesis and Evaluation of Glucosyl-, Acyl- and Silyl- Resveratrol Derivatives as Retinoprotective Agents: Piceid Octanoate Notably Delays Photoreceptor Degeneration in a Retinitis Pigmentosa Mouse Model
Source: Pharmaceuticals (Basel). 2024 Nov 5;17(11):1482. doi: 10.3390/ph17111482 (PMC11597447; doi:10.3390/ph17111482)
Supplement: Supplementary file 1 [file pharmaceuticals-17-01482-s001.zip › pharmaceuticals-3287101-supplementary.pdf]

Synthesis and Evaluation of Glucosyl-, Acyl- and Silyl- Resveratrol Derivatives as Retinoprotective Agents: Piceid Octanoate Notably Delays Photoreceptor Degeneration in a Retinitis Pigmentosa Mouse Model

Lourdes Valdés-Sánchez [1], Seyed Mohamadmehdi Moshtaghion [1,†], Estefanía Caballano-Infantes [1,†], Pablo Peñalver [2], Rosario Rodríguez-Ruiz [2], José Luis González-Alfonso [3], Francisco José Plou[3], Tom Desmet [4], Juan C. Morales [2,*] and Francisco J. Díaz-Corrales [1,*]

INDEX

Supplementary Figures S2-S7

^{1}H, ^{13}C NMR, and HRMS spectra for all new compounds S8-S19

Supplementary Figures

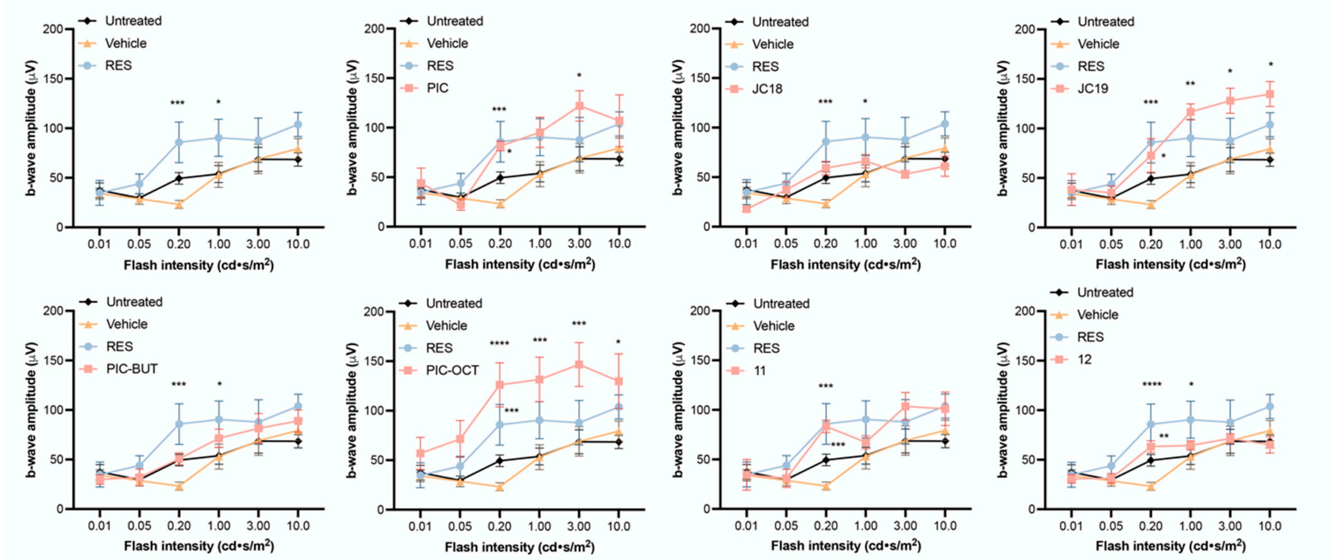

Figure S1. ERG quantification of b-wave amplitude of rd10 mice under dark-adapted conditions. The scotopic vision was evaluated in wild type (WT) and rd10 mice untreated or treated with 5% DMSO (vehicle) or with the different compounds (RES, PIC, JC18, JC19, PIC-BUT, PIC-OCT, **11** or **12**). The amplitude (µV) is shown. Different increasing flash intensities were tested (cd·s/m^2). Each graph represents the quantification of b-wave amplitude in different treated groups. The parametric two-way ANOVA test followed by Dunnett's multiple comparisons test evaluated statistically significant differences between vehicle and each of the other groups. A *p*-value less than 0.05 was considered statistically significant. *$p< 0.05$; **$p< 0.01$; ***$p< 0.001$; ****$p< 0.0001$.

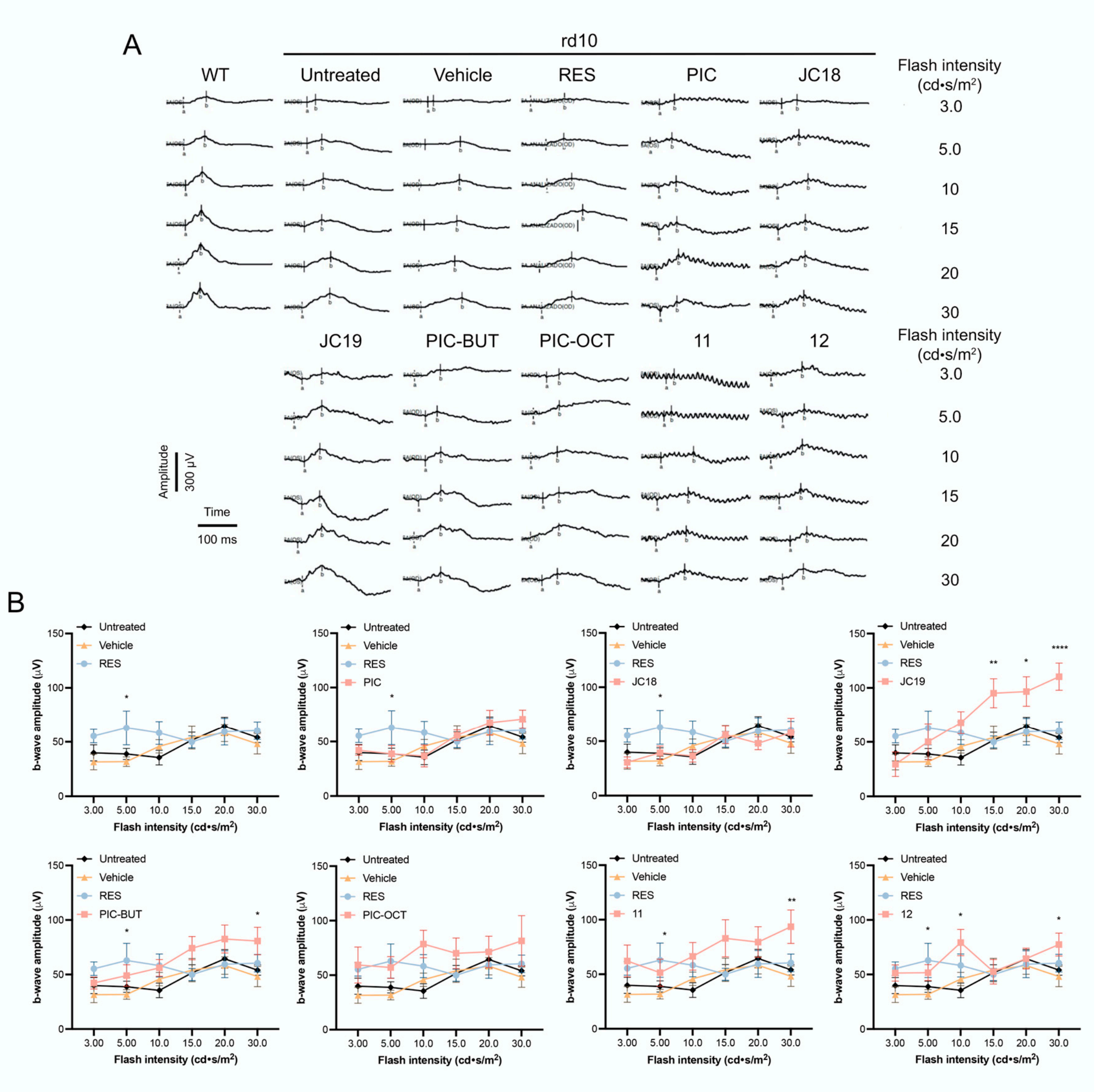

Figure S2. Representative ERG's traces under light-adapted conditions (**A**) and ERG quantification of b-wave amplitude of rd10 mice (**B**). The photopic vision was evaluated in wild type (WT) and rd10 mice untreated or treated with 5% DMSO (vehicle) or with the different compounds (RES, PIC, JC18, JC19, PIC-BUT, PIC-OCT, **11** or **12**). The amplitude (µV) and time (ms) scales are shown (A). Different increasing flash intensities were tested (cd·s/m^2). Each graph represents the quantification of b-wave amplitude in different treated groups (B). The parametric two-way ANOVA test followed by Dunn's multiple comparisons test evaluated statistically significant differences between vehicle and each of the other groups. A *p*-value less than 0.05 was considered statistically significant. **p*< 0.05; ***p*< 0.01; *****p*< 0.0001.

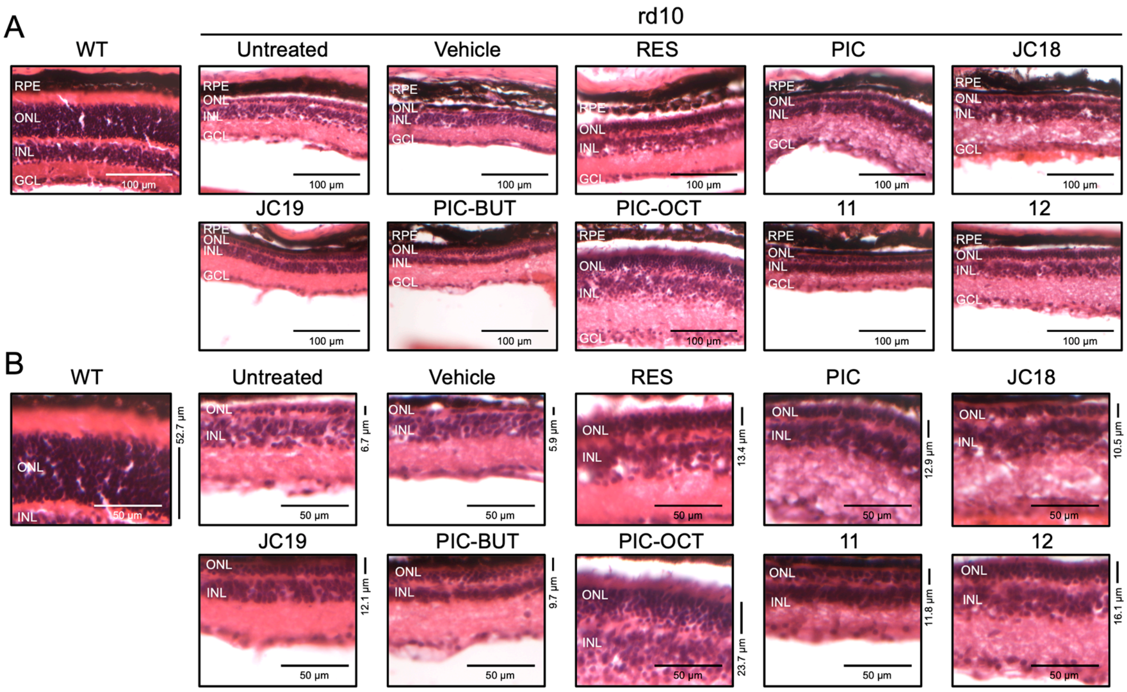

Figure S3. Outer nuclear layer (ONL) thickness measured in hematoxylin and eosin (H&E) stained retinal sections. Retinal sections of a wild type (WT) mouse and rd10 mice untreated or treated with 5% DMSO (vehicle) or with the different compounds (5mM RES, PIC, JC18, JC19, PIC-BUT, PIC-OCT, **11** or **12**). Mice were subretinal injected at P14 and then evaluated and euthanized by cervical dislocation at P28. The mouse eyes were quickly excised, fixed and stained with H&E. Scale bars in panel (**A**) represents 100 µm and in panel (**B**) represents 50 µm. Panel B shows the magnified images of the mouse retinas. PIC-OCT-treated mice showed the thickest ONL. RPE: retinal pigment epithelium; ONL: outer nuclear layer; INL: inner nuclear layer; GCL: ganglion cell layer.

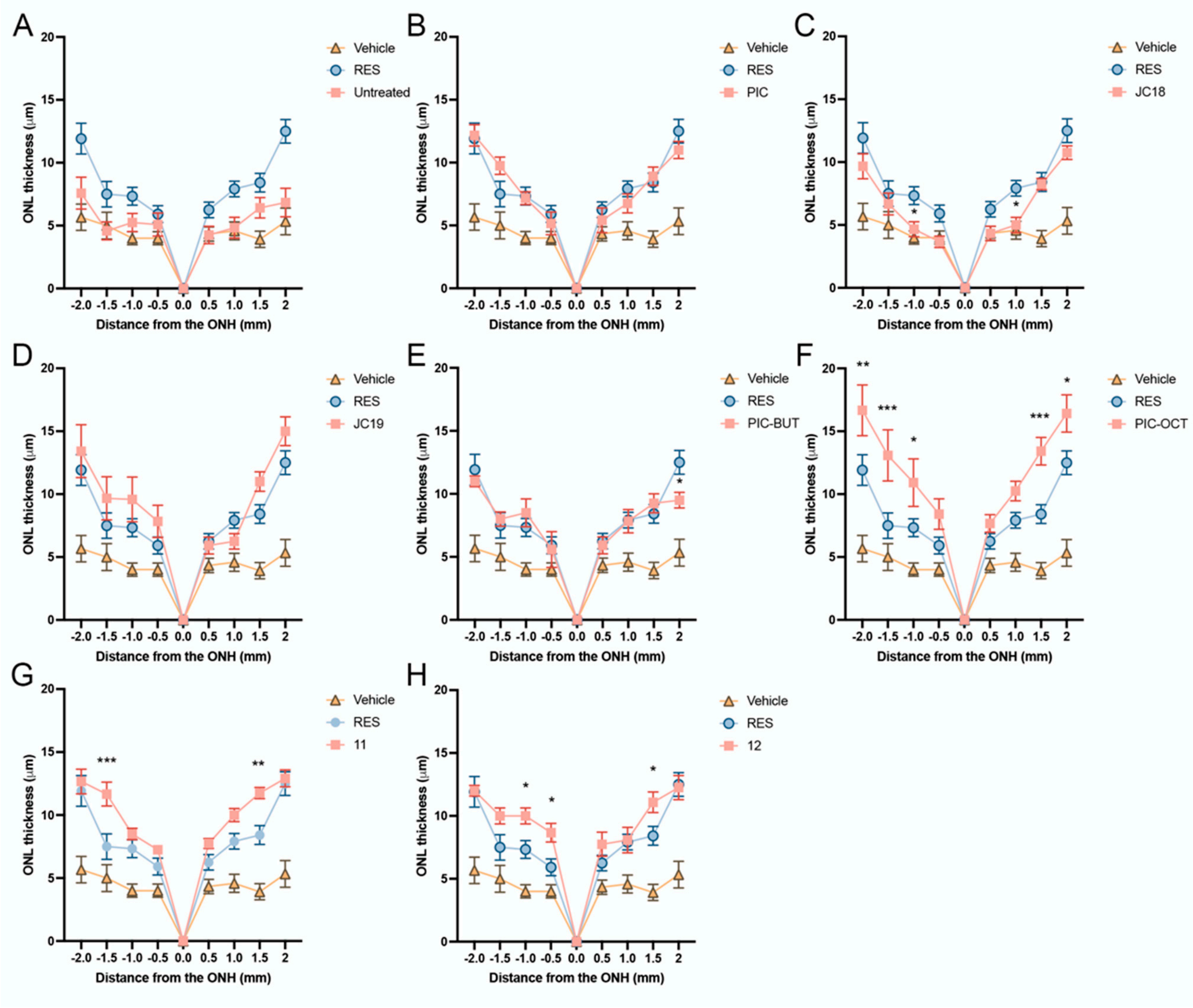

Figure S4. Quantification of outer nuclear layer (ONL) thickness. The ONL thickness of untreated rd10 mouse retinas, or treated with vehicle or with the different compounds (RES, PIC, JC18, JC19, PIC-BUT, PIC-OCT, **11** or **12**) 15 days after subretinal injections are shown (**A-H**). The graphs represent the mean ± SEM of ONL thickness in sagittal retinal sections of untreated or treated rd10 mice (**A-H**). The ONL thickness were measured at -2, -1.5, -1, -0.5, 0, 0.5, 1, 1.5, and 2 mm from the optic nerved head (ONH). The parametric two-way ANOVA test followed by Dunnett's multiple comparisons test evaluated statistically significant differences between vehicle, untreated and RES (**A**), and vehicle, RES and each of the other groups (**B-H**). A *p*-value less than 0.05 was considered statistically significant. *$p < 0.05$; **$p < 0.01$; ***$p < 0.001$.

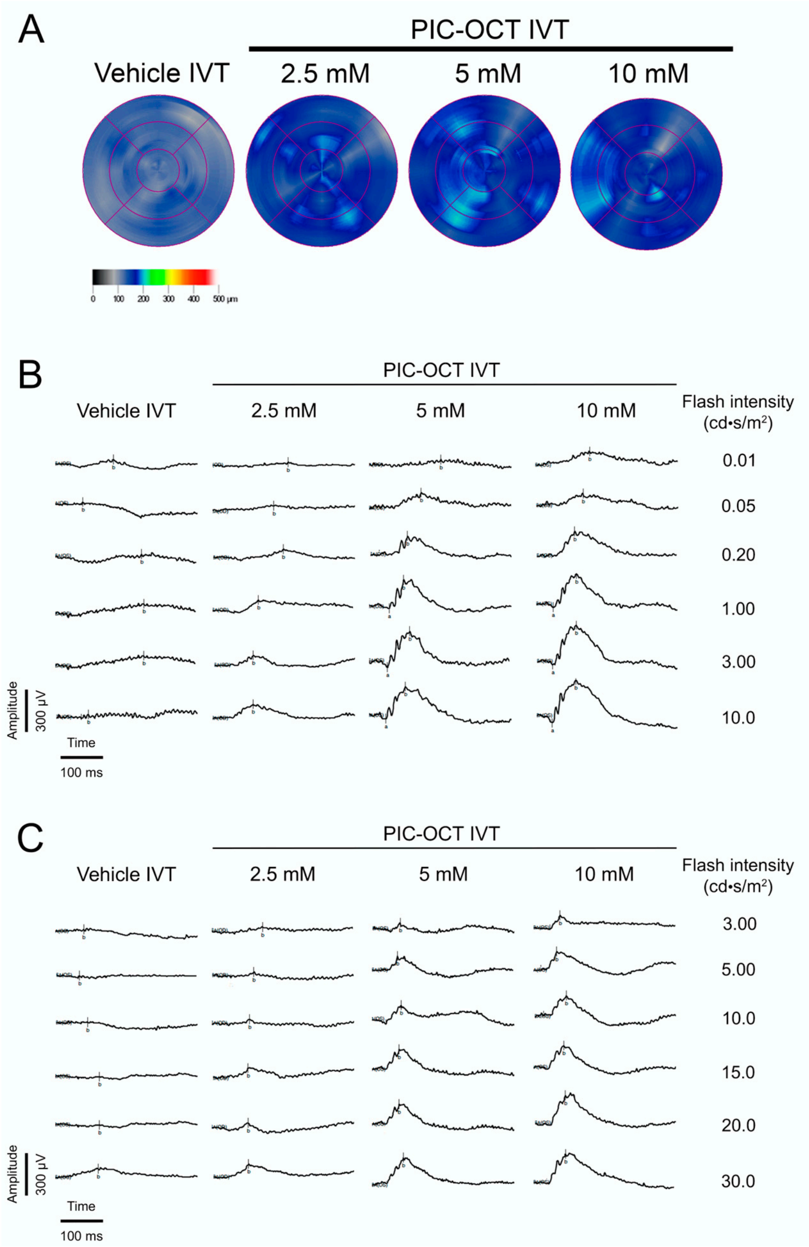

Figure S5. Retinal thickness and electroretinogram (ERG) traces of rd10 mice treated with different PIC-OCT doses. Retinal maps obtained by optical coherence tomography (OCT) scans of rd10 mice or treated intravitreal (IVT) injections of 5% DMSO (vehicle), or 2.5, 5 and 10 mM piceid octanoate (PIC-OCT) (**A**). Representative images of each group are shown. The colorimetric scale represents the retinal thickness in µm. Six radial scans were measured to construct the retinal maps. Representative ERG's traces under dark- (**B**) and light-adapted conditions (**C**). ERG quantification of b-wave amplitude of rd10 mice (**B**). The amplitude (µV) and time (ms) scales are shown. Different increasing flash intensities were tested (cd·s/m^2).

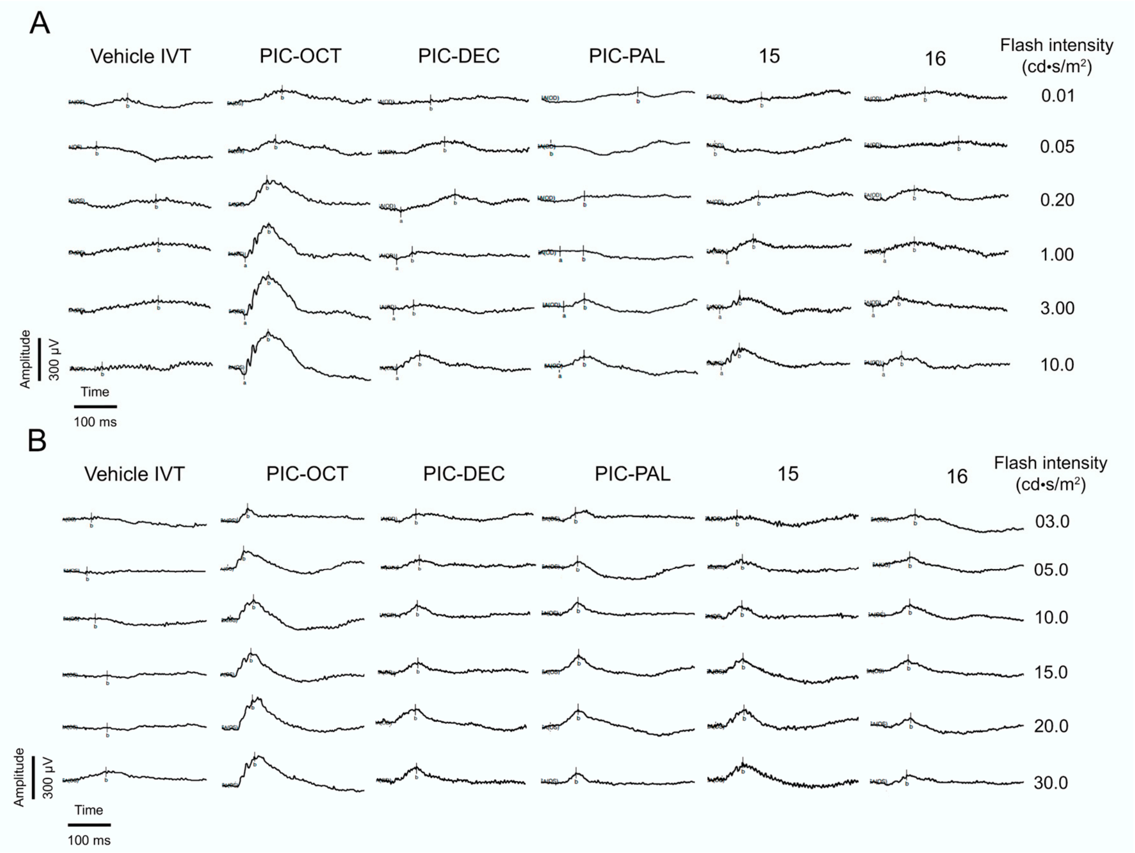

Figure S6. Electroretinogram (ERG) traces of rd10 mice treated with different PIC-OCT doses. Retinal maps obtained by optical coherence tomography (OCT) scans of rd10 mice or treated intravitreal (IVT) injections of 5% DMSO (vehicle), or piceid octanoate (PIC-OCT), and compounds PIC-DEC, PIC-PAL, **15** and **16**. Representative ERG's traces under dark-adapted (**A**) and light-adapted conditions (**B**). The amplitude (μV) and time (ms) scales are shown. Different increasing flash intensities were tested (cd·s/m^2).

^{1}H, ^{13}C NMR, and HRMS spectra for all new compounds

((2R,3S,4S,5R,6S)-3,4,5-trihydroxy-6-(3-hydroxy-5-((E)-4-hydroxystyryl)phenoxy)tetrahydro-2H-pyran-2-yl)methyl decanoate (13)

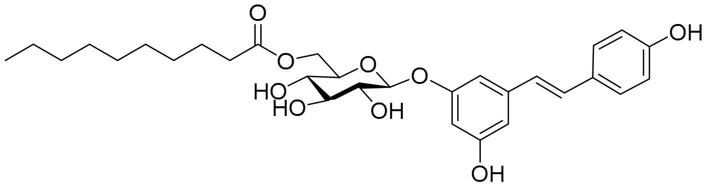

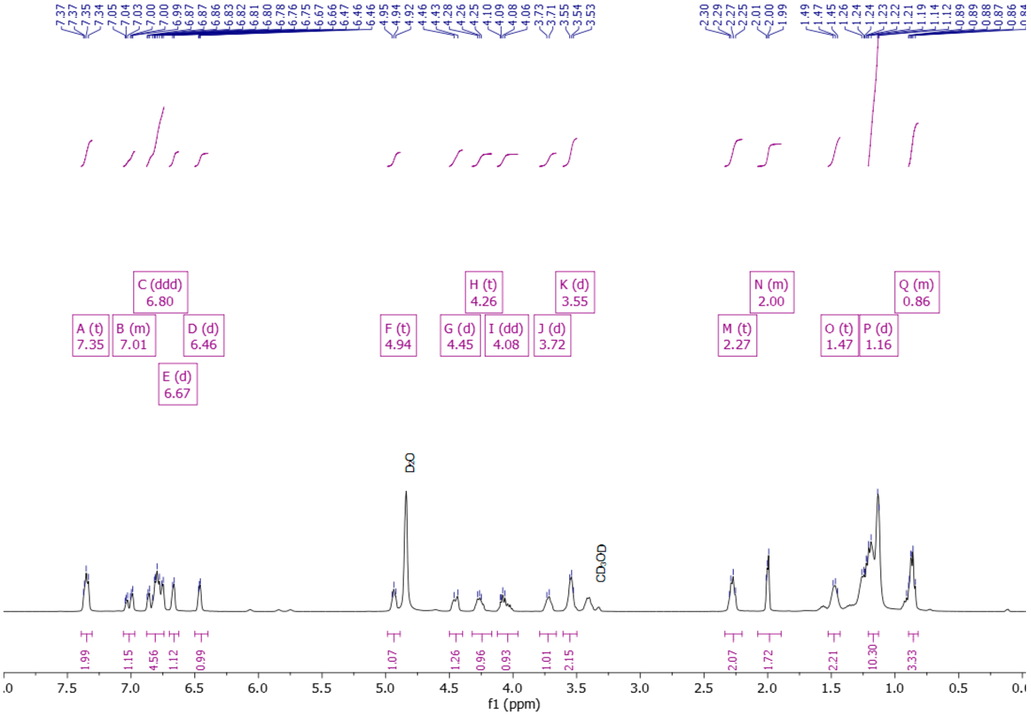

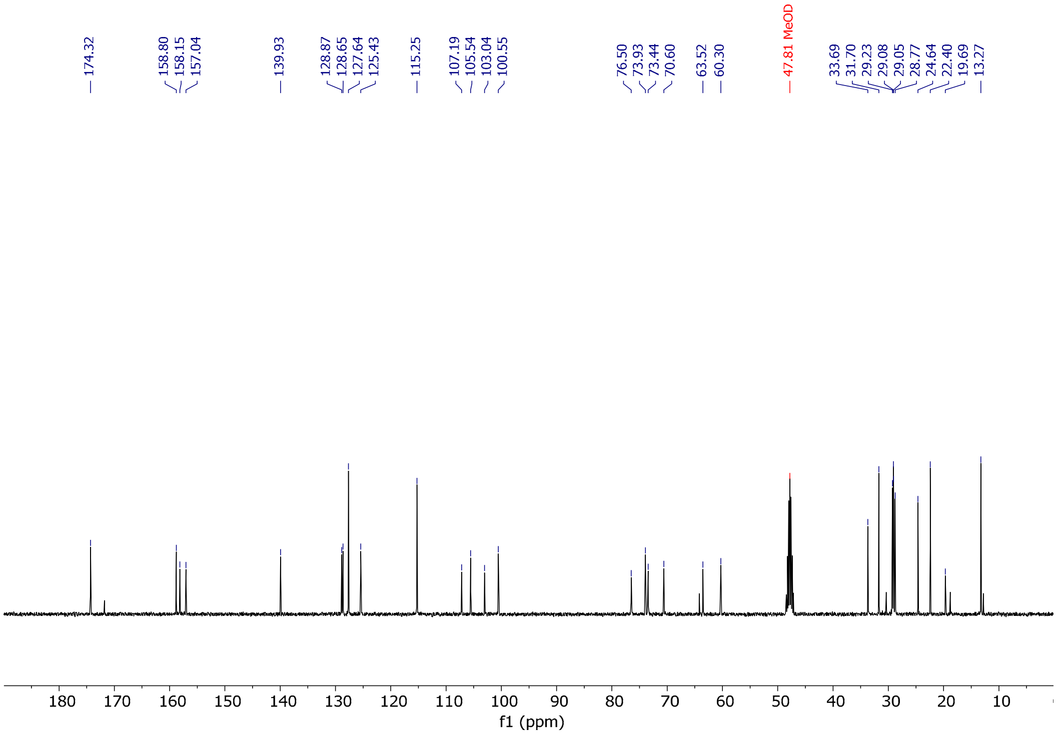

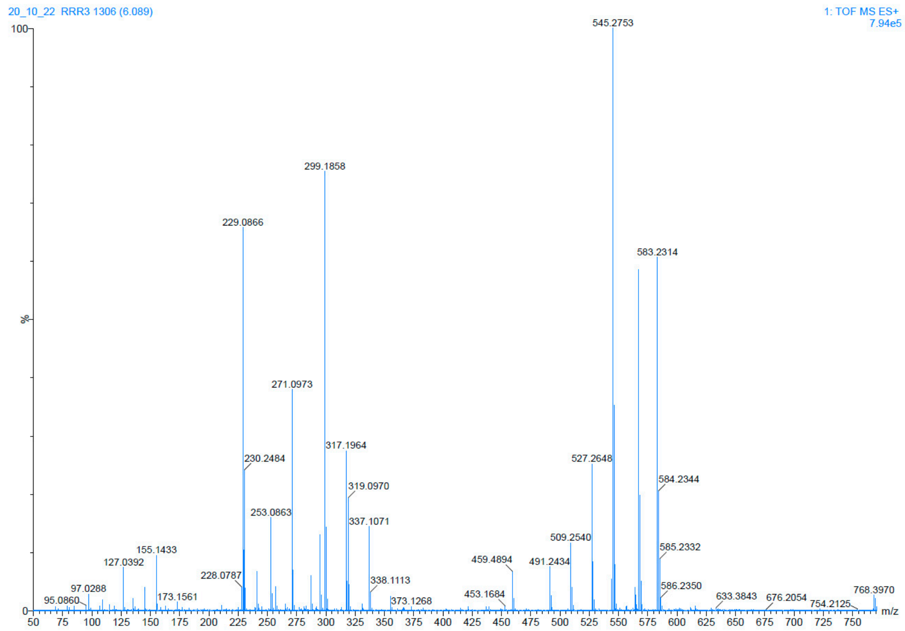

20_10_22 RRR3 1306 (6.089)
1: TOF MS ES+
7.94e5
545.2753
299.1858
229.0866
583.2314
271.0973
317.1964
230.2484
527.2648
584.2344
319.0970
253.0863
337.1071
509.2540
155.1433
585.2332
127.0392
459.4894
491.2434
228.0787
338.1113
97.0288
586.2350
173.1561
453.1684
633.3843
676.2054
768.3970
95.0860
373.1268
754.2125
m/z
%

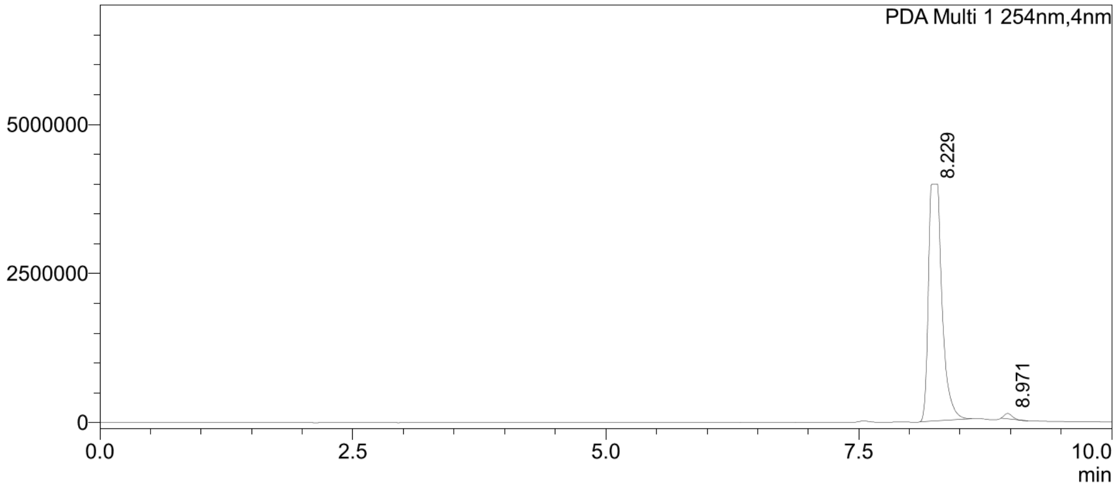

PDA Multi 1 254nm,4nm
5000000
2500000
0
8.229
8.971
0.0
2.5
5.0
7.5
10.0
min

((2R,3S,4S,5R,6R)-3,4,5-trihydroxy-6-(3-hydroxy-5-((E)-4-hydroxystyryl)phenoxy)tetrahydro-2H-pyran-2-yl)methyl octanoate (15)

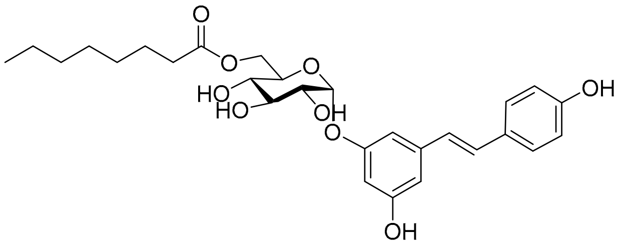

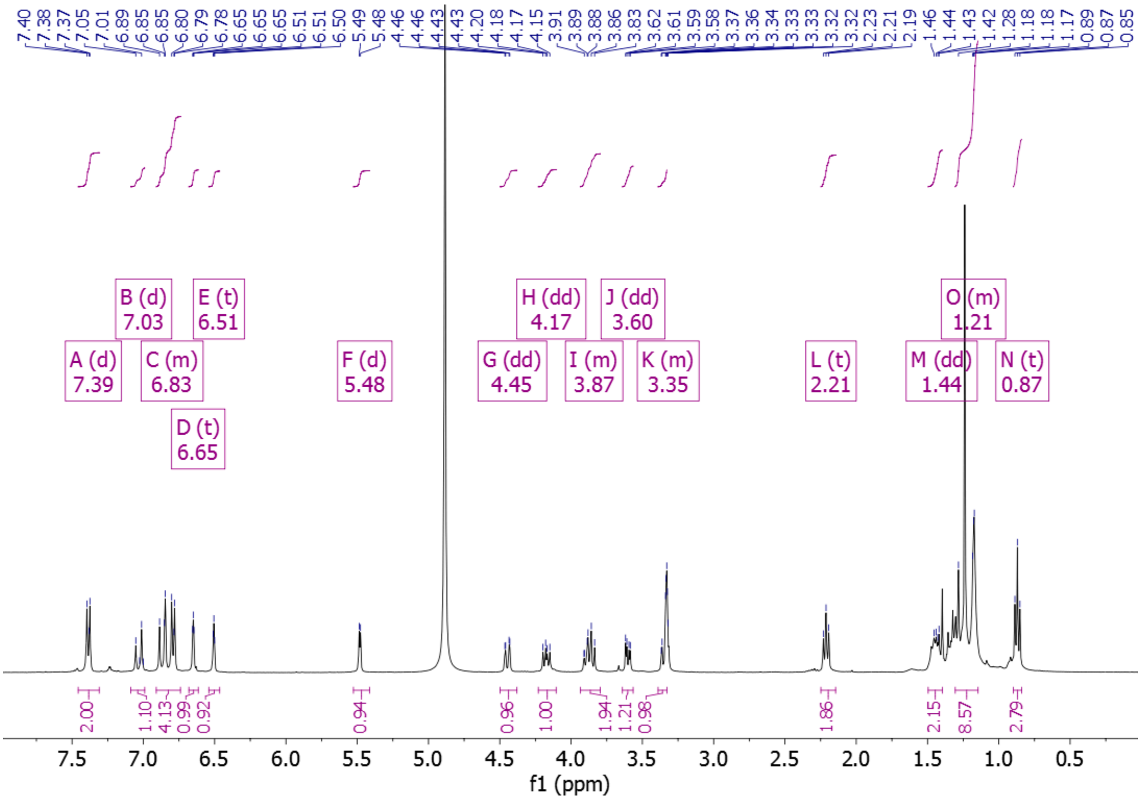

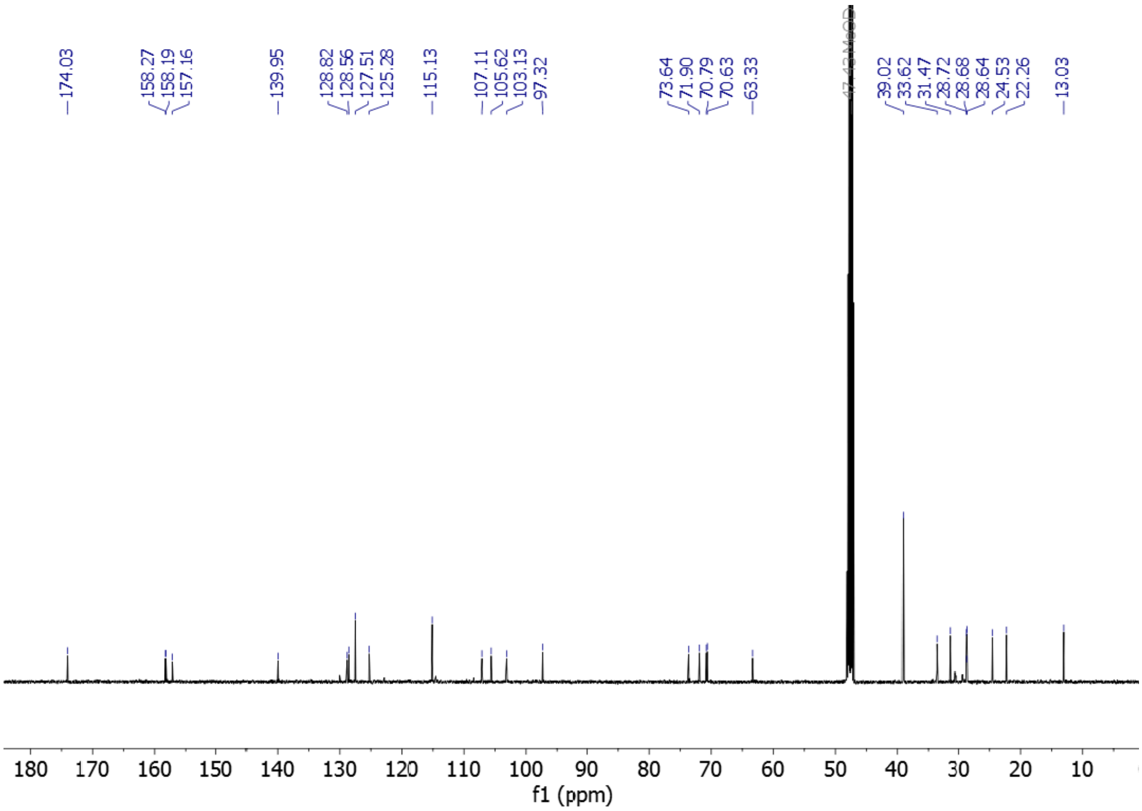
174.03
158.27
158.19
157.16
139.95
128.82
128.56
127.51
125.28
115.13
107.11
105.62
103.13
97.32
73.64
71.90
70.79
70.63
63.33
39.02
33.62
31.47
28.72
28.68
28.64
24.53
22.26
13.03
180 170 160 150 140 130 120 110 100 90 80 70 60 50 40 30 20 10
f1 (ppm)

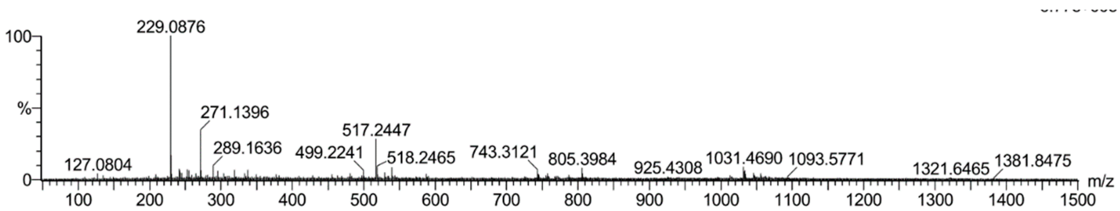
229.0876
271.1396
289.1636
517.2447
499.2241
518.2465
743.3121
805.3984
127.0804
925.4308
1031.4690
1093.5771
1321.6465
1381.8475
100
%
0
m/z
100 200 300 400 500 600 700 800 900 1000 1100 1200 1300 1400 1500

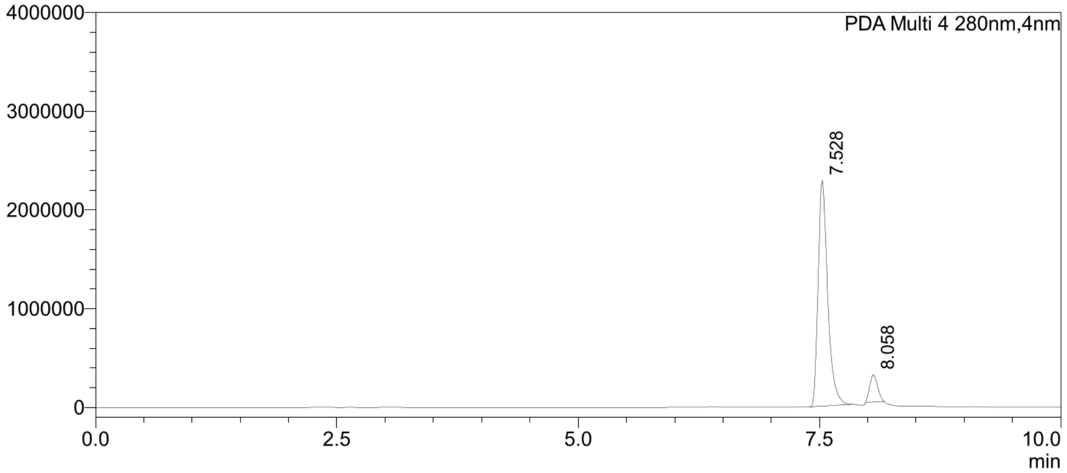
PDA Multi 4 280nm,4nm
7.528
8.058
4000000
3000000
2000000
1000000
0
0.0 2.5 5.0 7.5 10.0
min

(2R,3R,4S,5R)-2-(acetoxymethyl)-6-(4-((E)-3,5-bis((triisopropylsilyl)oxy)styryl)phenoxy)tetrahydro-2H-pyran-3,4,5-triyl triacetate. (3,5-diTIPS-4´-per-acetyl-glucosyl resveratrol, 18)

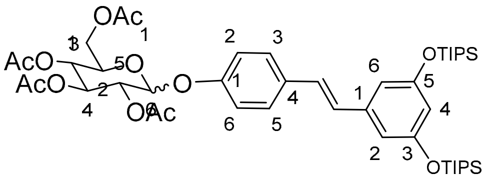

(2*R*,3*R*,4*S*,5*R*)-2-(acetoxymethyl)-6-(4-((*E*)-3,5-bis((triisopropylsilyl)oxy)styryl)phenoxy)tetrahydro-2*H*-pyran-3,4,5-triyl triacetate

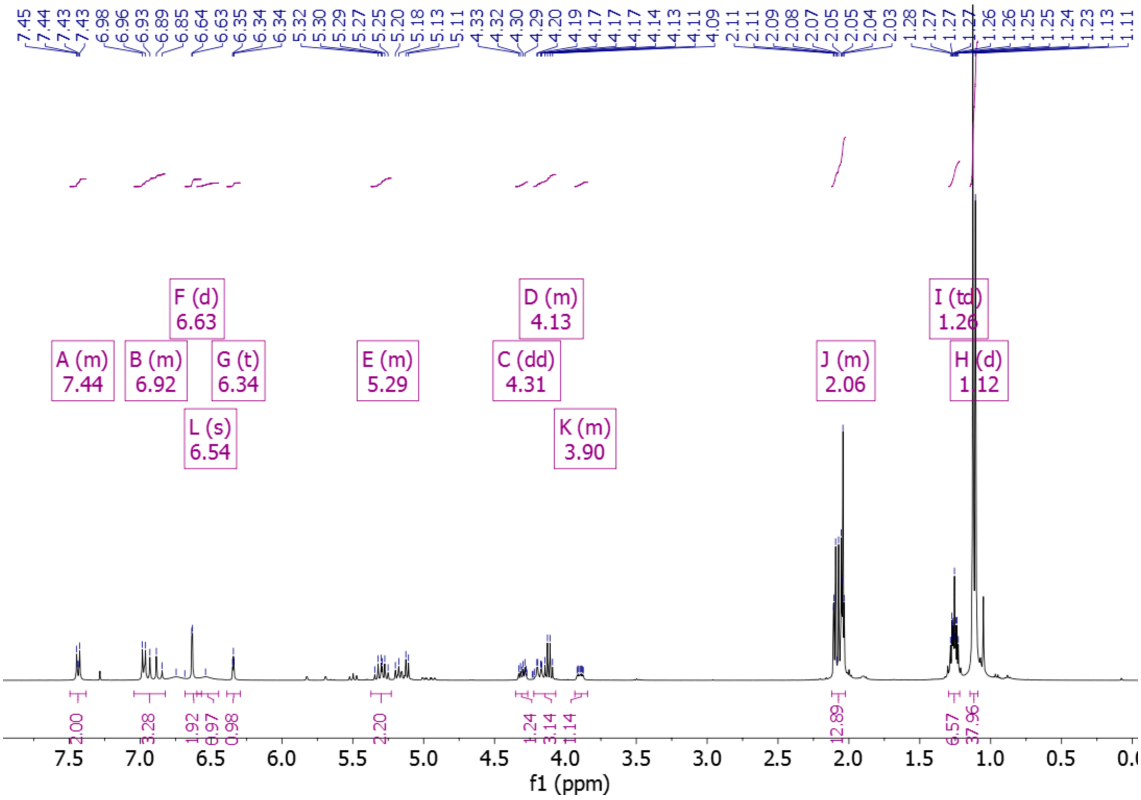

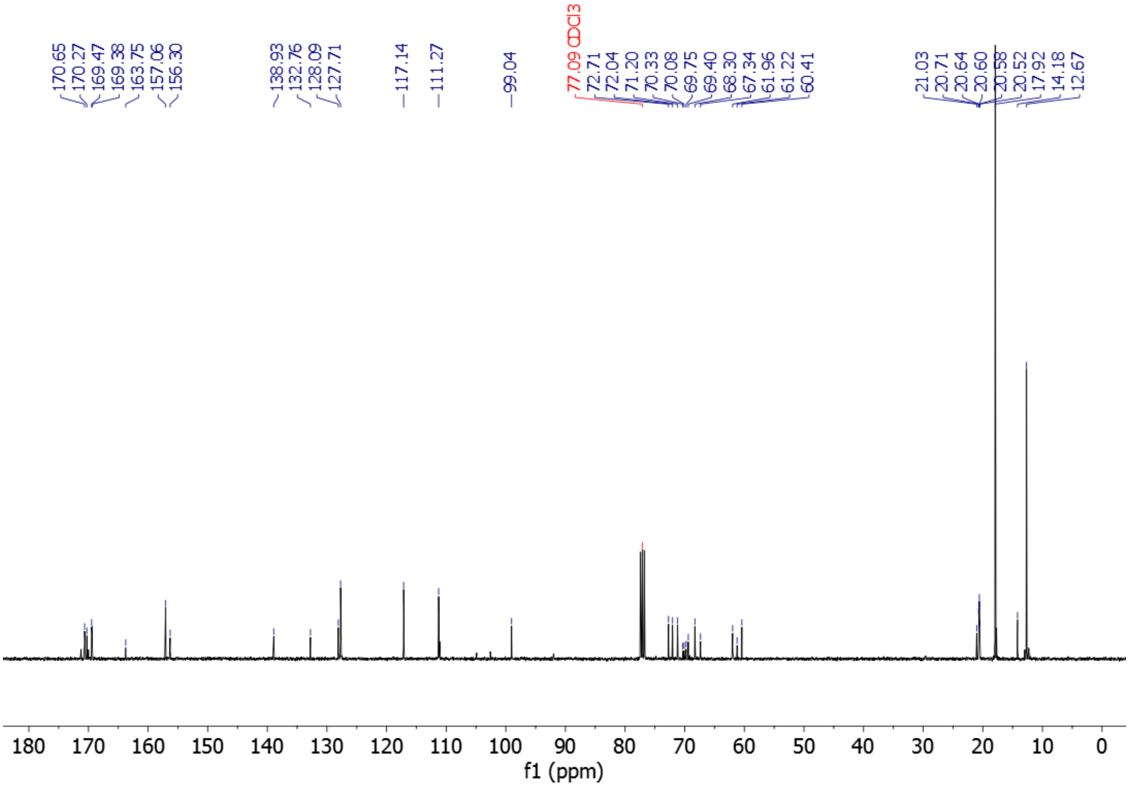
170.65
170.27
169.47
169.38
163.75
157.06
156.30
138.93
132.76
128.09
127.71
117.14
111.27
99.04
77.09 CDCl3
72.71
72.04
71.20
70.33
70.08
69.75
69.40
68.30
67.34
61.96
61.22
60.41
21.03
20.71
20.64
20.60
20.58
20.52
17.92
14.18
12.67
180 170 160 150 140 130 120 110 100 90 80 70 60 50 40 30 20 10 0
f1 (ppm)

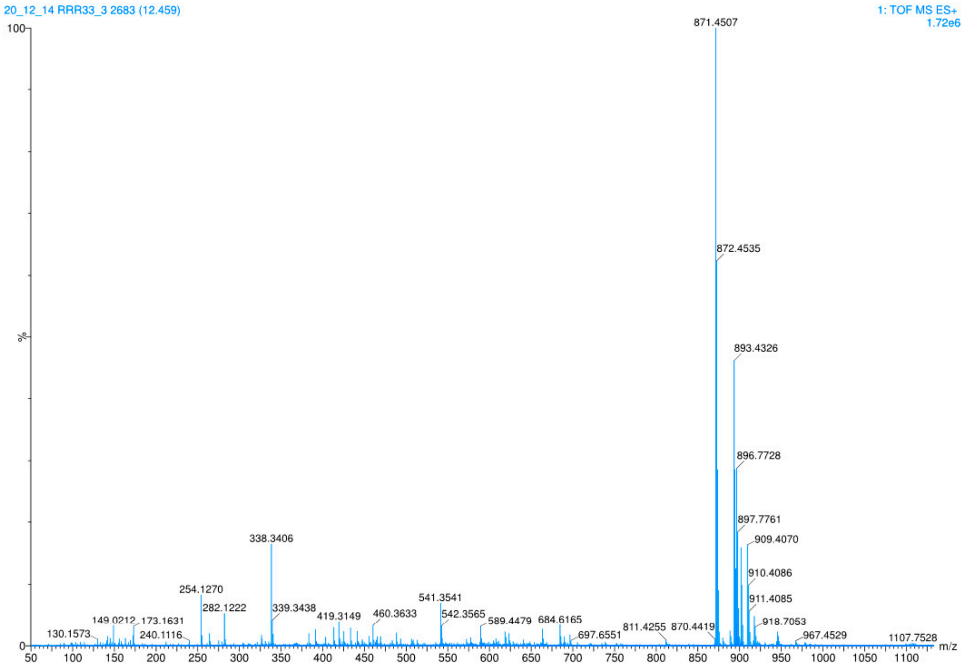
20_12_14 RRR33_3 2683 (12.459)
1: TOF MS ES+
1.72e6
100
%
0
871.4507
872.4535
893.4326
896.7728
897.7761
909.4070
910.4086
911.4085
918.7053
967.4529
1107.7528
338.3406
339.3438
254.1270
282.1222
240.1116
149.0212
173.1631
130.1573
419.3149
460.3633
541.3541
542.3565
589.4479
684.6165
697.6551
811.4255
870.4419
50 100 150 200 250 300 350 400 450 500 550 600 650 700 750 800 850 900 950 1000 1050 1100
m/z

(3R,4S,5S,6R)-2-(4-((E)-3,5-bis((triisopropylsilyl)oxy)styryl)phenoxy)-6-hydroxymethyl)-tetrahydro- 2H-pyran-3,4,5-triol. (3,5-diTIPS-4´-glucosyl resveratrol, 19)

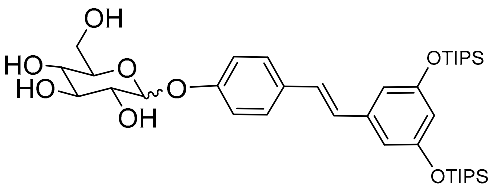

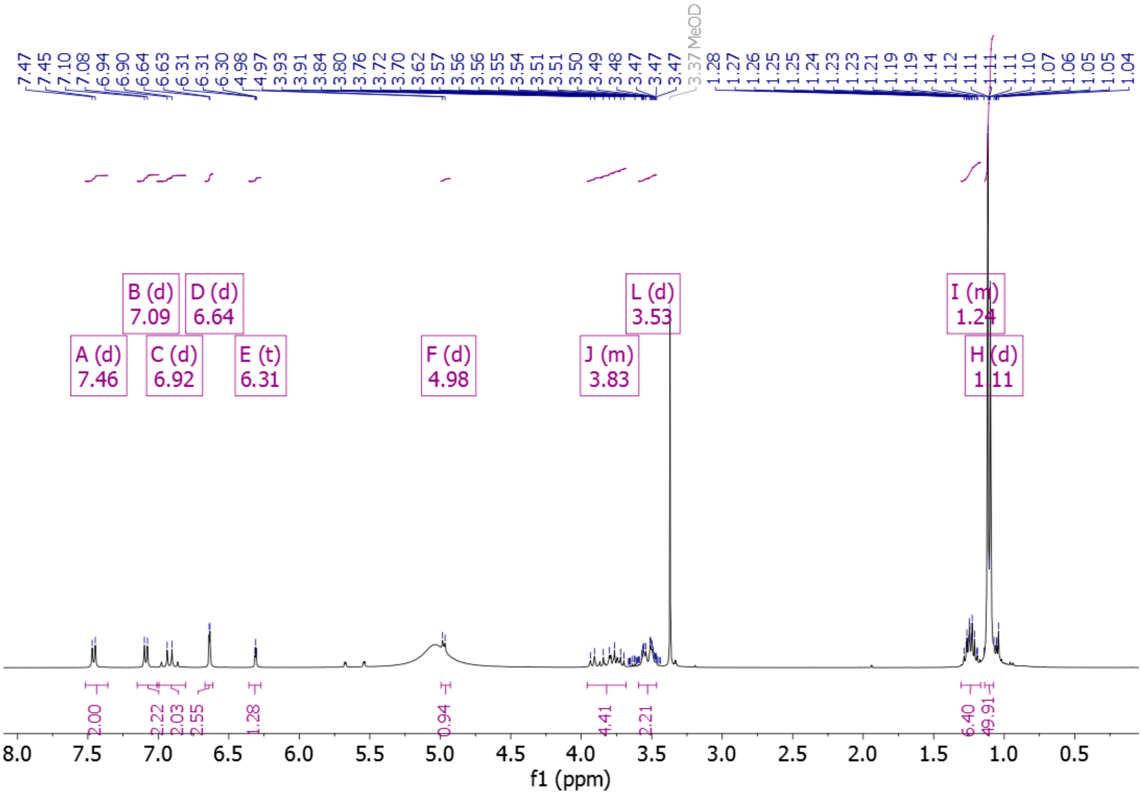

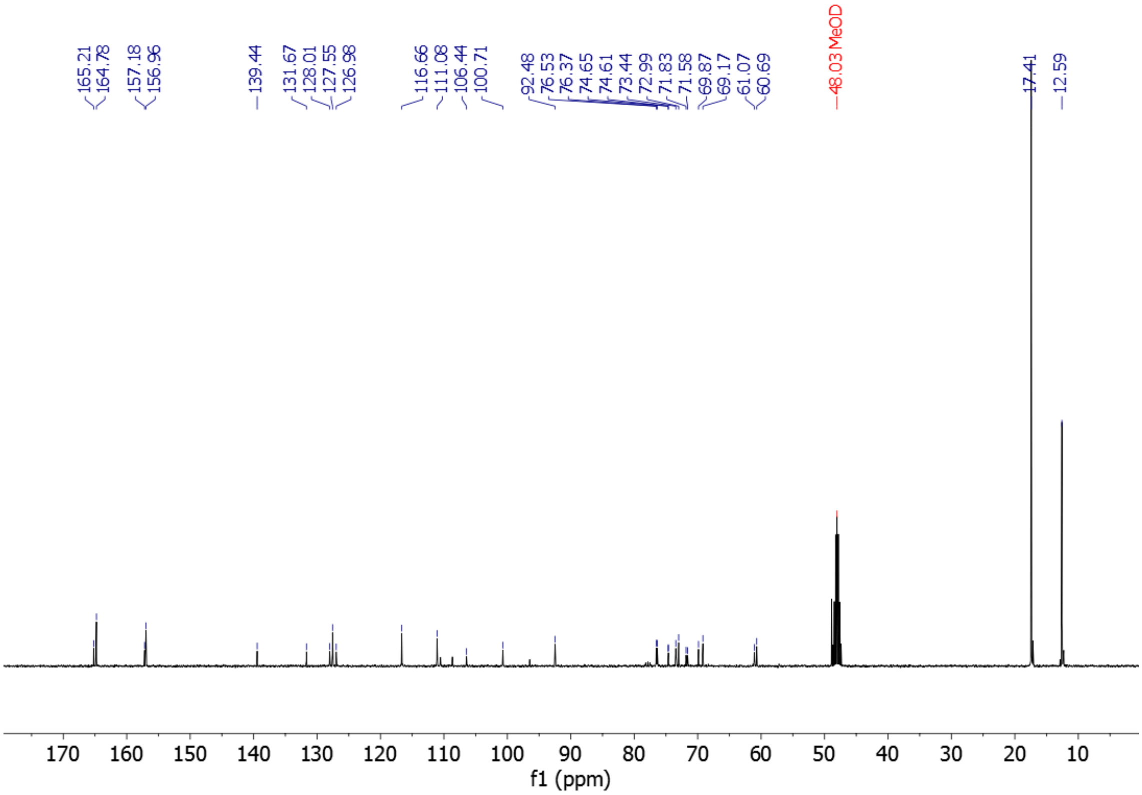
165.21
164.78
157.18
156.96
139.44
131.67
128.01
127.55
126.98
116.66
111.08
106.44
100.71
92.48
76.53
76.37
74.65
74.61
73.44
72.99
71.83
71.58
69.87
69.17
61.07
60.69
48.03 MeOD
17.41
12.59
f1 (ppm)

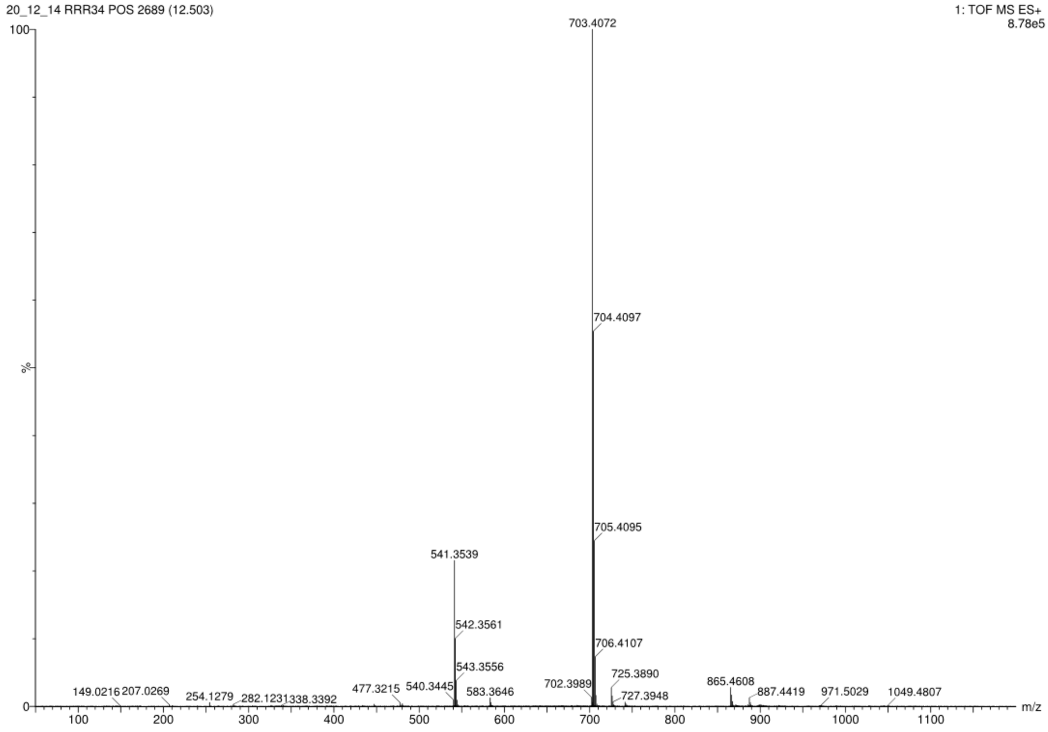
20_12_14 RRR34 POS 2689 (12.503)
1: TOF MS ES+
8.78e5
703.4072
704.4097
705.4095
706.4107
541.3539
542.3561
543.3556
540.3445
725.3890
727.3948
702.3989
865.4608
887.4419
971.5029
1049.4807
583.3646
477.3215
338.3392
282.1231
254.1279
207.0269
149.0216
%
m/z

((2R,3S,4S,5R)-6-(4-((E)-3,5-bis((triisopropylsilyl)oxy)styryl)phenoxy)-3,4,5-trihydroxytetrahydro-2H-pyran-2-yl)methyl octanoate. (3,5-diTIPS-4´- (6’’-octanoyl)-glucosyl resveratrol, 20)

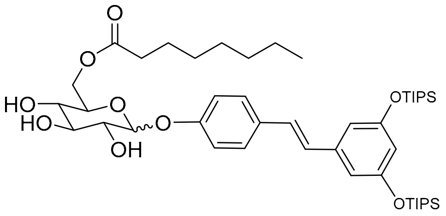

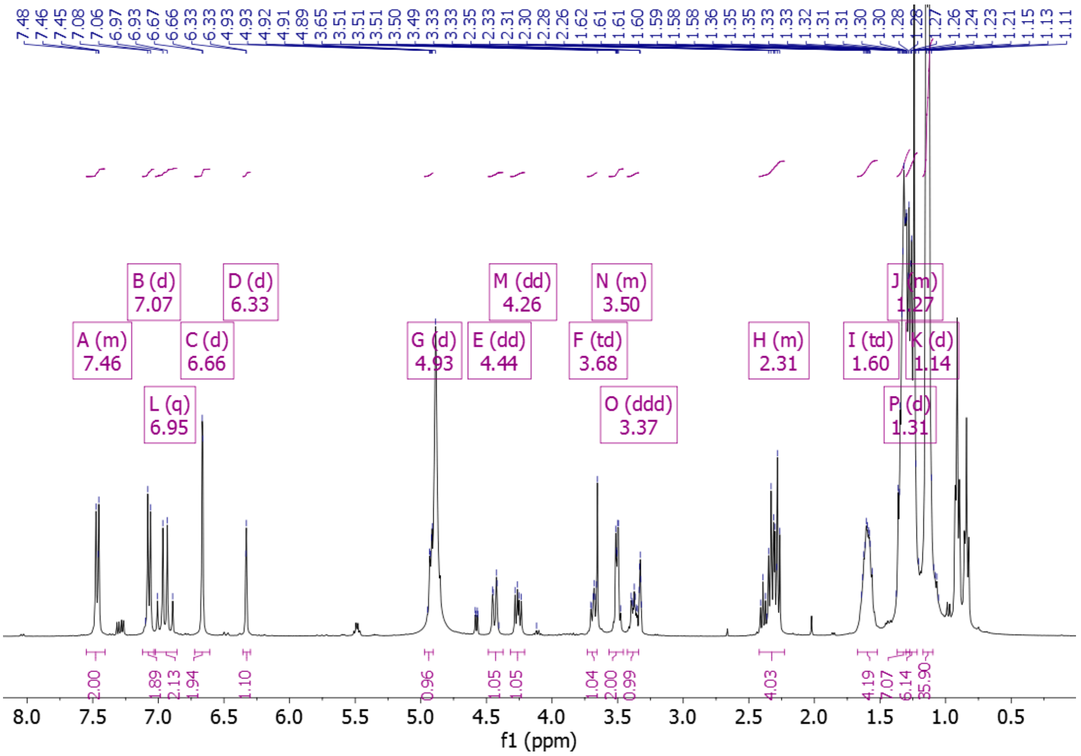

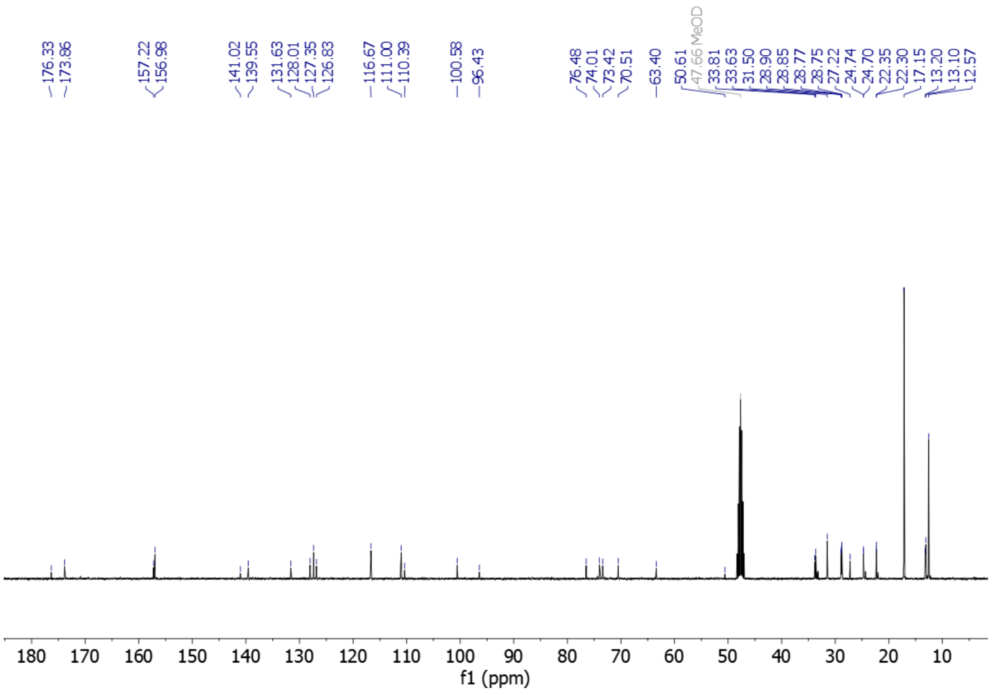

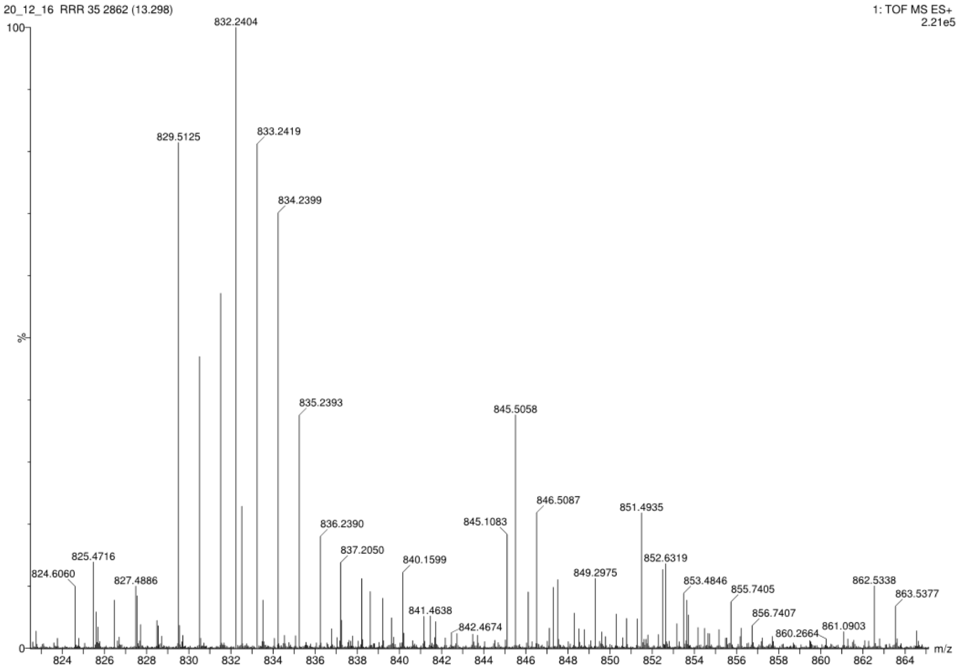
20_12_16 RRR 35 2862 (13.298)
1: TOF MS ES+
2.21e5
832.2404
829.5125
833.2419
834.2399
835.2393
845.5058
846.5087
851.4935
845.1083
836.2390
837.2050
840.1599
825.4716
824.6060
827.4886
849.2975
852.6319
853.4846
855.7405
862.5338
863.5377
841.4638
856.7407
842.4674
860.2664
861.0903
m/z
%

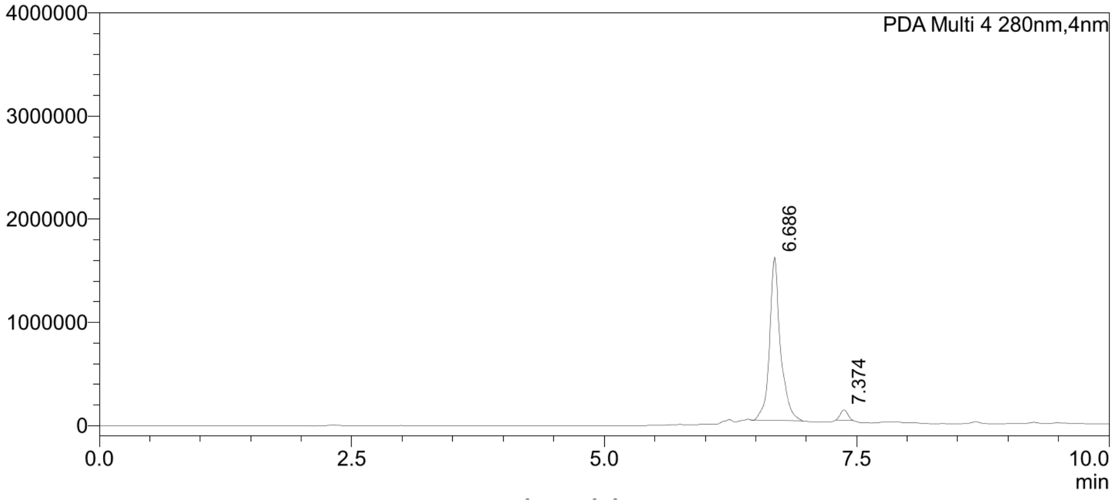
PDA Multi 4 280nm,4nm
6.686
7.374
min

((2R,3S,4S,5R)-6-(4-((E)-3,5-dihydroxystyryl)phenoxy)-3,4,5-trihydroxytetrahydro-2H-pyran-2-yl)methyl octanoate. (4´- (6’’-octanoyl)-glucosyl resveratrol, 16)

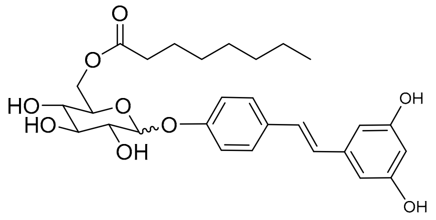

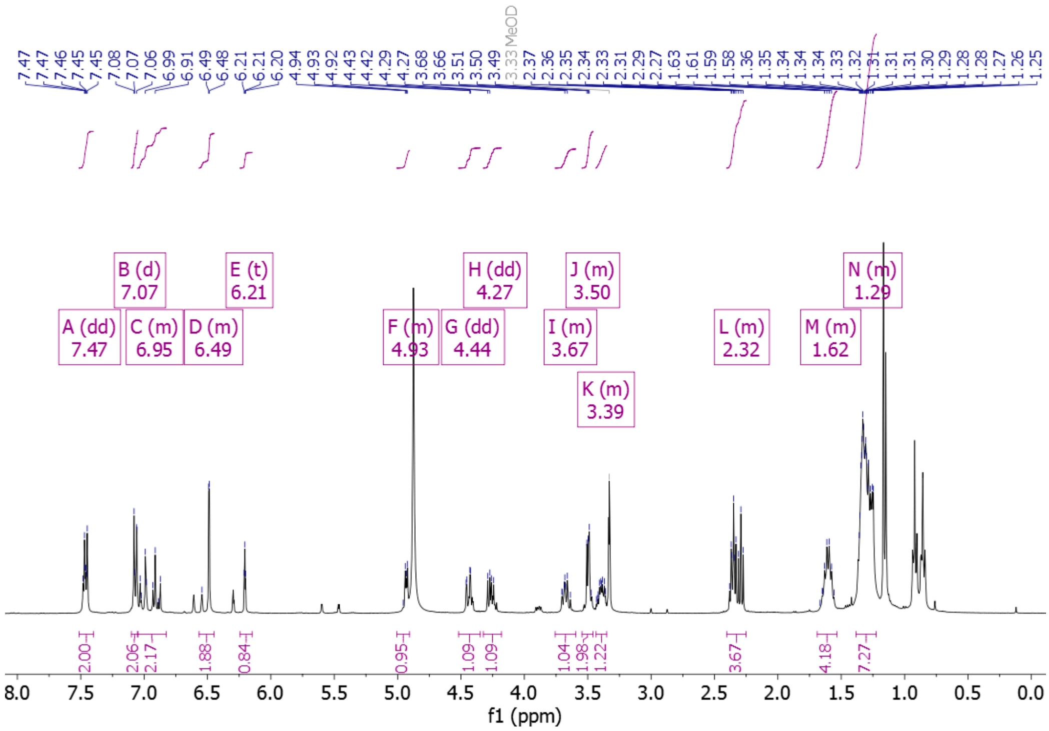

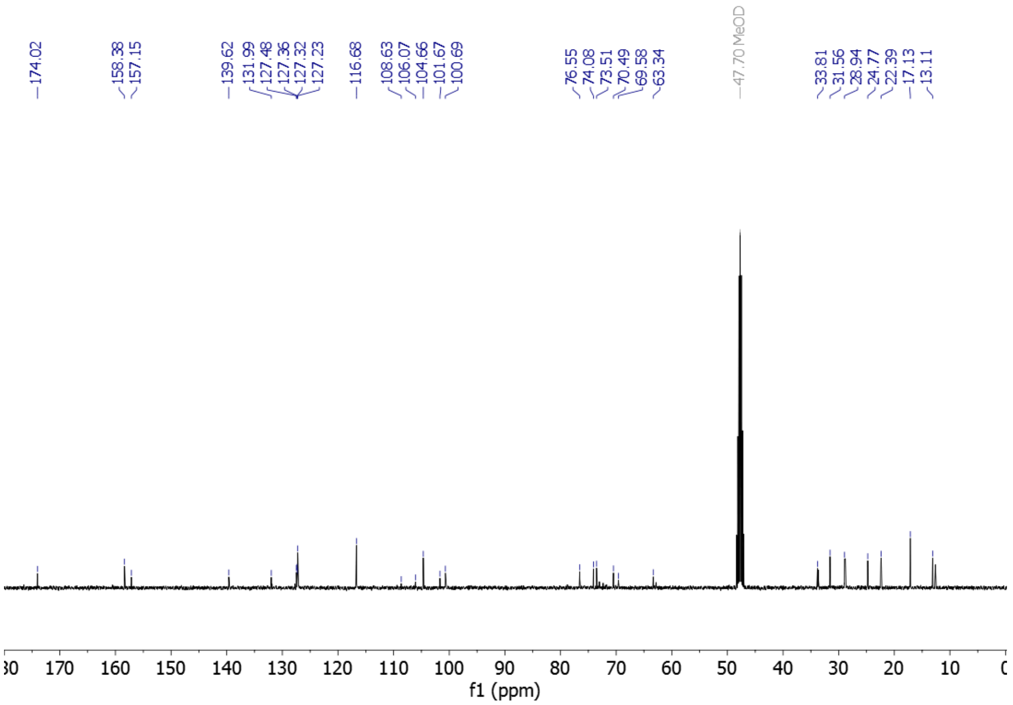

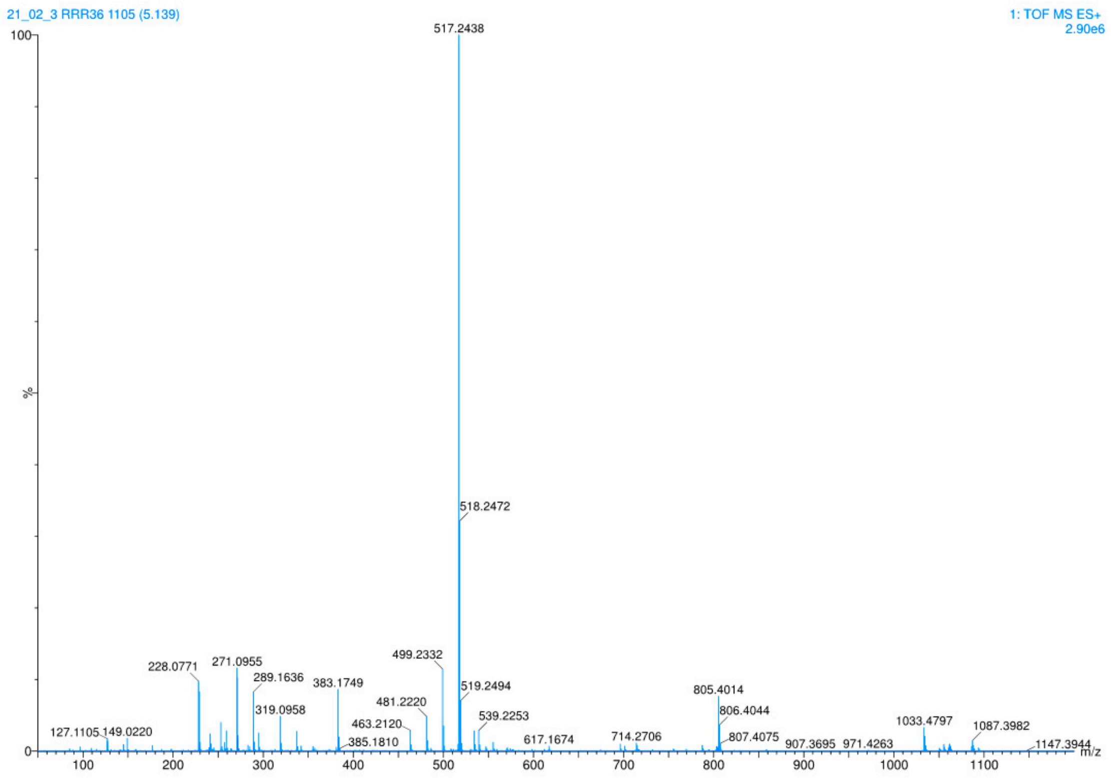
21_02_3 RRR36 1105 (5.139)
1: TOF MS ES+
2.90e6
517.2438
518.2472
499.2332
519.2494
228.0771
271.0955
289.1636
319.0958
383.1749
481.2220
463.2120
385.1810
539.2253
127.1105
149.0220
617.1674
714.2706
805.4014
806.4044
807.4075
907.3695
971.4263
1033.4797
1087.3982
1147.3944
%
m/z

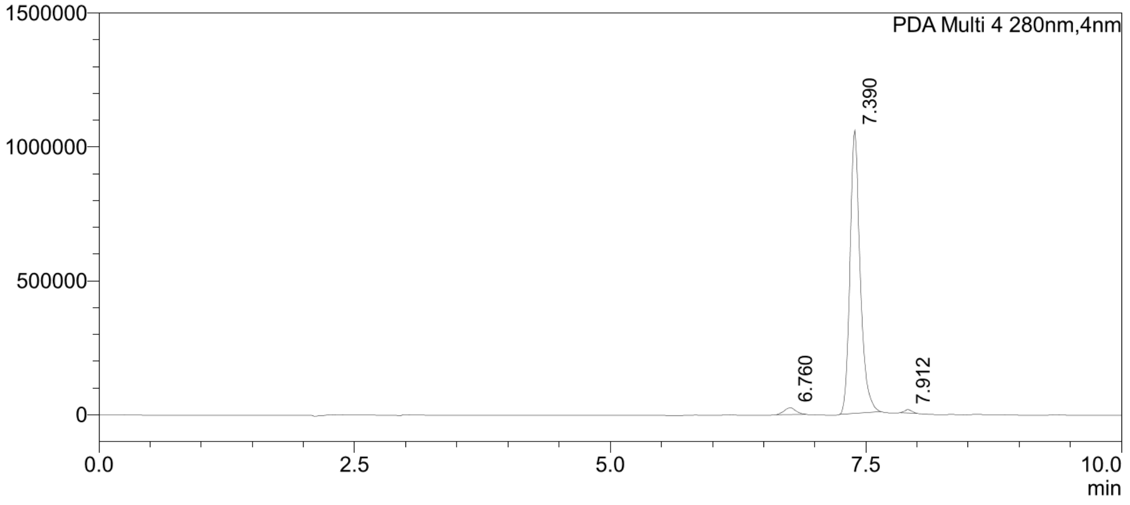
PDA Multi 4 280nm,4nm
7.390
6.760
7.912
min
